# Colorectal Cancer Screening within Colonoscopy Capacity Constraints: Can FIT-Based Programs Save More Lives by Trading off More Sensitive Test Cutoffs against Longer Screening Intervals?

**DOI:** 10.1177/23814683221097064

**Published:** 2022-05-07

**Authors:** Ethna McFerran, James F. O’Mahony, Steffie Naber, Linda Sharp, Ann G. Zauber, Iris Lansdorp-Vogelaar, Frank Kee

**Affiliations:** Queen’s University Belfast, Centre for Public Health, Institute of Clinical Sciences, Royal Victoria Hospital, Grosvenor Road, Belfast, UK; Centre for Health Policy and Management, Trinity College Dublin, The University of Dublin, Dublin, Ireland; Statistics Netherlands, the Netherlands; Newcastle University, Newcastle, UK; Department of Biostatistics, Memorial Sloan Kettering Cancer Center, New York, NY, USA; Department of Public Health of Erasmus MC, Rotterdam, the Netherlands; Centre for Public Health, Queen’s University Belfast, Belfast, UK

**Keywords:** colonoscopy capacity, colorectal cancer screening, FIT, optimization

## Abstract

**Introduction.** Colorectal cancer (CRC) prevention programs using fecal immunochemical testing (FIT) in screening rely on colonoscopy for secondary and surveillance testing. Colonoscopy capacity is an important constraint. Some European programs lack sufficient capacity to provide optimal screening intensity regarding age ranges, intervals, and FIT cutoffs. It is currently unclear how to optimize programs within colonoscopy capacity constraints. **Design.** Microsimulation modeling, using the MISCAN-Colon model, was used to determine if more effective CRC screening programs can be identified within constrained colonoscopy capacity. A total of 525 strategies were modeled and compared, varying 3 key screening parameters: screening intervals, age ranges, and FIT cutoffs, including previously unevaluated 4- and 5-year screening intervals (using a lifetime horizon and 100% adherence). Results were compared with the policy decisions taken in Ireland to provide CRC screening within available colonoscopy capacity. Outcomes estimated net costs, quality-adjusted life-years (QALYs), and required colonoscopies. The optimal strategies within finite colonoscopy capacity constraints were identified. **Results.** Combining a reduced FIT cutoff of 10 µg Hb/g, an extended screening interval of 4 y and an age range of 60–72 y requires 6% fewer colonoscopies, reduces net costs by 23% while preventing 15% more CRC deaths and saving 16% more QALYs relative to a strategy (FIT 40 µg Hb/g, 2-yearly, 60–70 year) approximating current policy. **Conclusion.** Previously overlooked longer screening intervals may optimize cancer prevention with finite colonoscopy capacity constraints. Changes could save lives, reduce costs, and relieve colonoscopy capacity pressures. These findings are relevant to CRC screening programs across Europe that employ FIT-based testing, which face colonoscopy capacity constraints.

## Highlights

### What Is Already Known about This Subject?

Some colorectal cancer screening programs lack sufficient colonoscopy capacity to provide optimal screening intensity in terms of screening age ranges, intervals, and FIT cutoffs. It is currently unclear how to optimize programs within colonoscopy capacity constraints.

### What Are the New Findings?

Longer screening intervals, previously not widely considered, when accompanied by more sensitive FIT cutoff thresholds, may help balance optimal cancer prevention with finite colonoscopy capacity constraints.

### How Might It Affect Clinical Practice in the Foreseeable Future?

In our case study, more lives and health services costs could be saved within existing colonoscopy capacity constraints if a lengthening of the screening interval was traded off against an increase in the screening age range and accompanied by the adoption of a more sensitive FIT cutoff. However, much larger increases in diagnostic capacity than currently planned appear warranted to realize the full potential of colorectal cancer screening.

## Introduction

Colorectal cancer (CRC) is a common malignancy that kills approximately 800 000 people globally each year.^1^ Early detection improves survival, with survival rates of 90% for locally detected disease versus 13% when metastasized.^2^ Screening for CRC has been shown to reduce both incidence and mortality.^3^ CRC screening is cost-effective when offered at an appropriate intensity.^4,5^

The advent of population-based CRC screening is relatively recent, with 14 European Union (EU) states adopting screening only after 2009. Organized CRC screening programs in Europe commonly use fecal-based tests such as the guaiac fecal occult blood tests (gFOBT) or fecal immunochemical testing (FIT).^6^ As of 2015, 20 of 28 EU member states were in various stages of implementing population-based CRC screening (Appendix Table 1).^7^ Recent reports show that more than half of these use FIT.^8^ Although the most common screening interval was every 2 year, there are significant differences in FIT thresholds in use, ranging from 6 to 180 µg of haemoglobin per gram of feces (µg Hb/g).

Programs using fecal-based primary screening typically use colonoscopy for secondary diagnostic testing of those with positive screening tests as well as within alternative routes of referrals and for post-treatment surveillance. Insufficient colonoscopy capacity can constrain what intensity and population coverage of CRC screening is feasible.^9,10^ Consequently, colonoscopy capacity imposes limits on how many lives can be saved through CRC screening.

The effectiveness and cost-effectiveness of population CRC screening varies with the breadth of the screening age range and length of the screening interval. In the case of FIT-based testing, effectiveness and cost-effectiveness also vary with the test cutoff used to determine positivity.^11^ Reducing the FIT cutoff improves sensitivity at the cost of reduced specificity. Shorter screening intervals, wider screening age ranges, and lower FIT cutoffs all lead to increased colonoscopy requirements. Despite the increase in colonoscopies, lower FIT cutoffs are generally more cost-effective.^12^

Most cost-effectiveness analyses (CEAs) of CRC screening do not consider the binding colonoscopy capacity constraints. Some studies have, however, shown how finite capacity might be best used in the Netherlands and Canada.^9,13,14^ The objective of this study is to further explore the potential for improved effectiveness and cost-effectiveness within a capacity-constrained system. In particular, while most existing CRC screening CEAs have explored screening intervals between 1 and 3 year,^15–19^ this analysis aims to investigate the potential of longer screening intervals to enhance screening effectiveness within colonoscopy constraints. It uses the example of the policy changes made in the Irish CRC screening program as a case study to investigate what alternative strategies could improve population health outcomes.

### Case Study

The challenges facing European CRC screening are demonstrated by the case of the Irish CRC screening program. It serves as a useful example as the screening strategy was initially specified following a health economic analysis and has been modified since in response to colonoscopy capacity constraints. The initial health technology assessment (HTA) that informed the establishment of Ireland’s CRC screening program was conducted in 2009.^20,21^ It simulated comparisons of FIT, gFOBT, and once-off sigmoidoscopy. FIT and gFOBT were considered over a limited selection of age ranges at 1 screening interval of 2 y. The FIT test performance characteristics were derived from pooled analyses and employed a single FIT cutoff of 20 µg Hb/g of feces, equivalent to 100 nanograms of hemoglobin per milliliter of buffer (ng Hb/mL).^21,22^ The HTA found that biennial FIT between the ages of 55 and 74 y was the optimally effective and cost-effective strategy. However, insufficient colonoscopy capacity prevented the implementation of this strategy and prompted further analyses.^23,24^ These analyses suggested a narrower age range as one way to operate within existing colonoscopy capacity.^24^ These subsequent assessments did not examine varying the screening interval or FIT cutoffs.

The program was launched in October 2012 with biennial screening offered between ages 60 and 69 y at a cutoff of 20 µg Hb/g (FIT 100 ng Hb/mL). The stated intention was to expand to the initially planned 55- to 74-year age range as colonoscopy capacity developed.^25^ In practice, colonoscopy capacity constraints persisted, leading to a second policy change in early 2014. The FIT cutoff was increased from 20 to 45 µg Hb/g (100 to 225 ng Hb/mL).^23^ Although adopting a higher cutoff would improve specificity and ease colonoscopy demand, the loss of sensitivity would reduce screening effectiveness.^26^ Restoring the 55- to 74-year age range was recently restated as a policy objective, but reducing the FIT threshold was not.^27^

## Methods

We used a microsimulation model to estimate the costs and effects of a broad range of FIT-based screening strategies. We simulated the policy choices made to date to address colonoscopy capacity constraints and attempted to find alternative policies that are feasible given these constraints but offered greater effectiveness and cost-effectiveness.

We used the MISCAN-Colon model to simulate multiple screening strategies in a single birth cohort of average-risk individuals, until death. This established microsimulation model was developed at the Erasmus University Medical Center.^28^ The model used the parameterization as applied in a Dutch population and was not calibrated for the Irish population. Its underlying structure and parameters have been subjected to comparative evaluations against other CRC screening models.^29^ An overview of the model natural history, as applied in our analyses of CRC screening, in this and in other studies,^9,30^ is publicly available.^28^ Extensive model validation of CRC predictions has been undertaken based on international trial data,^29,31^ along with detailed analysis of the role of assumptions regarding adenoma progression.^32^

MISCAN-Colon simulates the life histories of individuals who may develop 1 or more adenomas. These adenomas may progress from small (≤5 mm) to medium (6 to 9 mm) to large (≥10 mm) lesions. Some adenomas will develop into preclinical cancer, which may then progress through stages I to IV. Symptomatic presentation of CRC is possible at any stage. Survival after clinical diagnosis is determined by the stage at diagnosis, the localization of the cancer, and the person’s age.

Screening alters some of the simulated life histories through the detection and removal of adenomas or the detection of cancer earlier than a clinical presentation, potentially leading to improved prognosis due to earlier treatment.^2^ However, screening can also result in serious complications, including perforation^33^ and overdiagnosis and overtreatment of CRC (i.e., the detection and treatment of cancer that would not otherwise progress to affect quality of life or life expectancy).^34^ By comparing all simulated life histories with and without screening, MISCAN-Colon estimates the cost and effectiveness of the alternative screening strategies. Although patients were not involved in this study because of the nature of the methods applied, this work seeks to advocate for their interests in the policy-practice interface.

### Test Characteristics

The FIT test characteristics (Table 1) were taken from published estimates.^35,36^ In the absence of a consistent source of test performance characteristics corresponding to the case study program cutoff of 45 µg Hb/g (225 ng Hb/mL),^21^ we used published estimates for 40 µg Hb/g (200 ng Hb/mL) as an approximation. Colonoscopy test characteristics are those applied routinely with MISCAN.^37^ The model assumes that 95% of all colonoscopies reach the cecum^38^ and that the remaining 5% are distributed uniformly over the colon and rectum.

**Table 1:** Test Characteristics within the Base Case and a Sensitivity Analysis

		Sensitivity per Lesion, %				
		Adenoma			CRC	
FIT Cutoff Level (µg Hb/g)^35 a^	Specificity (per Person, %)	≤5 mm	6–9 mm	≥10 mm	CRC Early Stage	CRC Late Stage
Base case test performance assumptions						
10	95.79	0.0	9.6	16.1	65.0	90.0
15	97.05	0.0	5.7	14.4	58.5	87.0
20	97.76	0.0	4.4	13.1	52.0	83.5
30	98.34	0.0	2.9	12.3	50.5	83.0
40	98.70	0.0	2.5	10.3	50.0	82.5
Colonoscopy^37^	100.00	75.0	85.0	95.0	95.0	95.0
Test performance assumptions within sensitivity analysis^36^						
20	92.00	0.0	4.4	42.0	33.0	
40	95.90	0.0	2.5	24.0	25.0	
Colonoscopy	100.00	77.0	77.0	98.0	98.0	

a According to the manufacturer, the OC-SENSOR delivers 10 mg of feces into 2.0 mL of buffer; thus, a test result of 100 ng hemoglobin per milliliter of buffer equals 20 µg hemoglobin per gram of feces.^22^

### Diagnostic Testing and Surveillance

The model assumes that diagnostic colonoscopy is offered after any positive FIT. If no adenomas or CRCs are found, individuals return to routine screening. Adenomas detected at colonoscopy are assumed to be removed by polypectomy, and individuals then enter colonoscopy-based surveillance following risk-based guidelines: with surveillance colonoscopy in 1- and 3-year intervals, in high risk (all lesions ≥10 mm) and intermediate risk (>2 lesions <10 mm), respectively,^39^ to a maximum age of 80 y. Low-risk cases (<3 adenomas <10 mm) are returned to routine FIT screening, based on customary practice.^40–42^ The model simulates total colonoscopy requirements for each strategy including those for (secondary) diagnostic testing, surveillance, and clinical presentations of the disease.

### Screening, Surveillance Strategies, and Attendance Assumptions

As our purpose was to broaden the scope for optimizing screening within colonoscopy capacity constraints, we simulated 525 screening strategies in addition to no screening. We modeled 5 FIT cutoffs of 10, 15, 20, 30, and 40 µg Hb/g (equivalent to 50, 75, 100, 150, and 200 ng Hb/mL). We considered intervals of 1, 2, 3, 4, and 5 y. In addition to the current program start and stop age of 60 and 70 year, respectively, we simulated screening start ages of 45, 50, 55, 60, 65, and 70 year, with stop ages of 70, 75, and 80 y or close approximations thereof depending on the screening interval (Table 2).

**Table 2:** Simulated Screening Strategy Characteristics

Strategy Characteristics	
Screening interval (y)	1/2/3/4/5
Start age (y)	45/50/55/60/65/70
Stop ages (y)	70/75/80
Fecal immunochemical testing cutoff levels (µg Hb/g)	10/15/20/30/40

All strategies were assessed in terms of the net cost and health effects measured in quality-adjusted life-years (QALYs) relative to no screening. Both costs and effects were discounted at 3% in accordance with the previous Dutch analyses on which our model is based.^13,43^ Assumed adherence was 100%. The model used a lifetime time horizon. The net cost assumptions included the costs of screening, diagnostic colonoscopy, surveillance, and any net changes in treatment costs due to early intervention (Table 3).

**Table 3:** Principal Model Assumptions

Variable	Base-Case Value		Sensitivity Analyses	
Discount rate	3%		1.5% or 5%	
Time horizon	Lifetime		N/A	
Adherence rate to all testing	100%		50% or 80%	
Fatal complication rate after colonoscopy	1 in 10,000		N/A	
Dwell time, average (interquartile range)	10.6 year, (5–14 year)^32^			
Incidence rate			Incidence was increased by 50% and reduced by 50%	
Complication rate of colonoscopy	0.24%		N/A	
FIT costs (€)				
Costs per invitation (organization and test kit)	14.85			
Costs per attendee (personnel and material for analysis	4.37			
Colonoscopy costs (€)				
Without polypectomy	303			
With polypectomy	393			
Cost of complications with colonoscopy	1250			
Treatment costs (€)^12^	Initial Treatment	Continuous Care	Terminal Care, Death of CRC	Terminal Care, Death of Other Cause
Stage 1	12,500	340	17,500	4400
Stage 2	17,000	340	17,500	4000
Stage 3	21,000	340	18,500	5200
Stage 4	25,000	340	25,000	14,000

In addition to this base-case analysis, we considered a series of 1-way sensitivity analyses that examined imperfect participation in primary FIT screening, assuming each individual has an 80% and 50% probability of participting in each screening round. We considered a low- and a high-incidence scenario in which the incidence of disease was decreased and increased by 50% relative to the base case, respectively; we also assessed results using low and high discount rates of 1.5% and 5%. We also considered a limited alternative scenario for the FIT cutoffs of 20 and 40 µg Hb/g in which we adopted the same test performance characteristics for FIT and colonoscopy as examined in another recent study of CRC screening within capacity constraints, as detailed in Table 1.^36^

We estimated the current colonoscopy capacity constraint in the case study program as the simulated lifetime colonoscopy demand of the current policy. This was the capacity required for a biennial FIT test in those aged 60 to 69 y with a FIT cutoff of 40 µg Hb/g. We also estimated the implied capacity constraint for the planned expansion of the age range to 55 to 75 y. We determined the optimally cost-effective strategies within the implied current and future capacity constraints. We used a cost-effectiveness threshold of €20 000/QALY to determine cost-effectiveness.^44^

The following “Results” section outlines the overall cost and effect estimates. We give a detailed description of the policy changes taken to date and their estimated outcomes. We then consider what policy alternatives exist within current colonoscopy capacity. Finally, we consider how the program might be optimally expanded beyond the current colonoscopy capacity.

## Results

An overview of all simulated strategies is presented in Supplementary Appendix Table 3, including the FIT cutoff, screening interval, and age range along with the estimated colonoscopy requirements, costs, effects, and total CRC deaths prevented. The current strategy requires 464 colonoscopies over the lifetime of 1000 individuals. While many strategies exceed current colonoscopy capacity (305 strategies), there are 220 that do not. Many strategies feasible within the colonoscopy constraint (*n* = 85) are more effective than the current strategy is. Some (*n* = 5) are cost saving relative to the current program.

**Figure 1:**
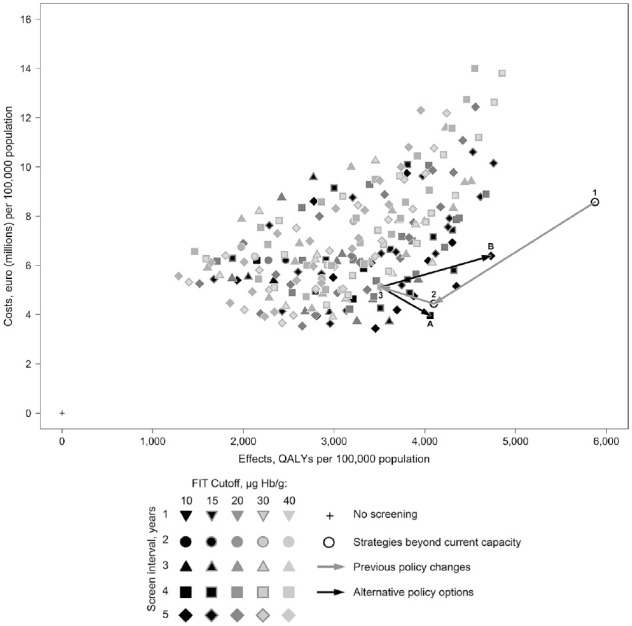
Past policy changes of an initial restriction in the screening age range (from points 1 to 2) and an increase in the fecal immunochemical testing cutoff (points 2 to the status quo of 3) and 2 alternative policies within current capacity of A: increasing effectiveness while not increasing cost; B: the optimally cost-effective strategy.

Figure 1 plots the screening strategies that are feasible within the implied capacity of the current screening strategy shown by point 3. This efficient set within this figure is exclusively composed of strategies with a FIT cutoff of 10 µg Hb/g (50 ng Hb/mL). This indicates that the lowest cutoff generally yields strategies that are more effective and less costly. The figure also illustrates previous policy changes and some future policy options. These are further detailed in Table 4. The originally HTA-recommended strategy (point 1) requires a colonoscopy capacity of more than twice the present strategy (1017 colonoscopies per 1000 persons), which would also incur greater costs and yield greater benefits than the status quo. Narrowing the age range to 60 to 69 y adopted at the program’s introduction in 2012 (point 2) reduced the colonoscopy requirements by almost half (662 colonoscopies per 1000 persons). The 2014 increase in the FIT cutoff to 45 µg Hb/g further reduces colonoscopy demand but also reduces effectiveness and modestly increases costs (point 3: current strategy).

**Table 4:** Summary of Policy Positions

Identifier	Strategy	Age Range (y)	Interval (y)	FIT Cut off (µg Hb/g)	QALYs per 1000	Cost (€) per 1000	Colonoscopies per 1000	Change in QALYs (%)^a^	Change in Costs (%)^a^	Change in Colonoscopies (%)^a^
1	Initial recommendation	55–75	2	20	59	85,748	1017	67	67	119
2	Age restriction	60–70	2	20	41	44,422	662	17	−13	43
3	Approximation of current strategy	60–70	2	40	35	51,201	464	REF	REF	REF
4	Planned age expansion	55–75	2	40	52	93,152	735	47	82	59
A	Max NHB with cost-saving	60–72	4	10	41	39,680	437	16	−23	−6
B	Max NHB within capacity	55–75	5	10	47	63,861	455	35	25	−2
C	Optimized (max NHB) with expanded capacity	50–74	4	10	58	95,271	707	66	86	52
D	Max overall net health benefit	50–80	1	10	92	215,284	3669	163	320	691

FIT, fecal immunochemical testing; NHB, net health benefit, at a cost-effectiveness threshold of €20,000/QALY; QALY, quality-adjusted life-year.

a Percentage change relative to the current strategy (strategy 3).

### Potential Policy Alternatives

Two potential policy alternatives to the status quo are illustrated in Figure 1. Both options A and B are within the current colonoscopy capacity and thus are now feasible. Option A uses a FIT cutoff of 10 µg Hb/g (50 ng Hb/mL) with a 4-year screening interval for those aged 60 to 72 y. It dominates the current policy, offering 16% more QALYs, 15% more CRC deaths prevented, 23% less costs, and requires 6% fewer colonoscopies relative to the current strategy, strategy 3. Option B is the optimally cost-effective currently feasible strategy. It uses a 10 µg Hb/g (FIT 50 ng Hb/mL) cutoff with a 5-year screening interval between ages 55 to 75 y. It provides an approximate 35% gain in QALYs, 29% more CRC deaths prevented, and a modest 2% reduction in colonoscopies relative to the current strategy but at a 25% cost increase.

**Figure 2:**
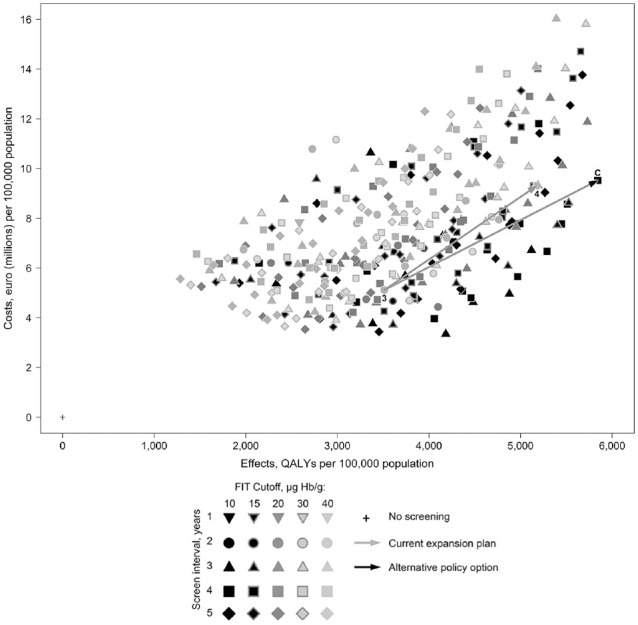
The current service expansion plan (from the status quo of point 3 to point 4 based on an expansion in the age range only) and the optimally effective and cost alternative within the implied increase in capacity at point C.

The current Irish national cancer strategy includes a policy commitment to restore the initially planned 55- to 74-year age range; however, this does not mention plans to change the screening interval or FIT cutoff.^27^ This policy is illustrated as point 4 in Figure 2. Figure 2 also included the other strategies that would be feasible in the colonoscopy capacity expansion of 59% relative to the status quo implied by point 4. Strategy C is an alternative policy using the same implied increased capacity. This uses a 10-µg Hb/g (FIT 50 ng Hb/mL) cutoff with a 4-year interval between ages 50 to 74 y. It would provide a 13% QALY gain relative to the planned age expansion (strategy 4) but would also be 2% more costly; it would, however, require 4% fewer colonoscopies than those predicted for the planned age expansion.

**Figure 3:**
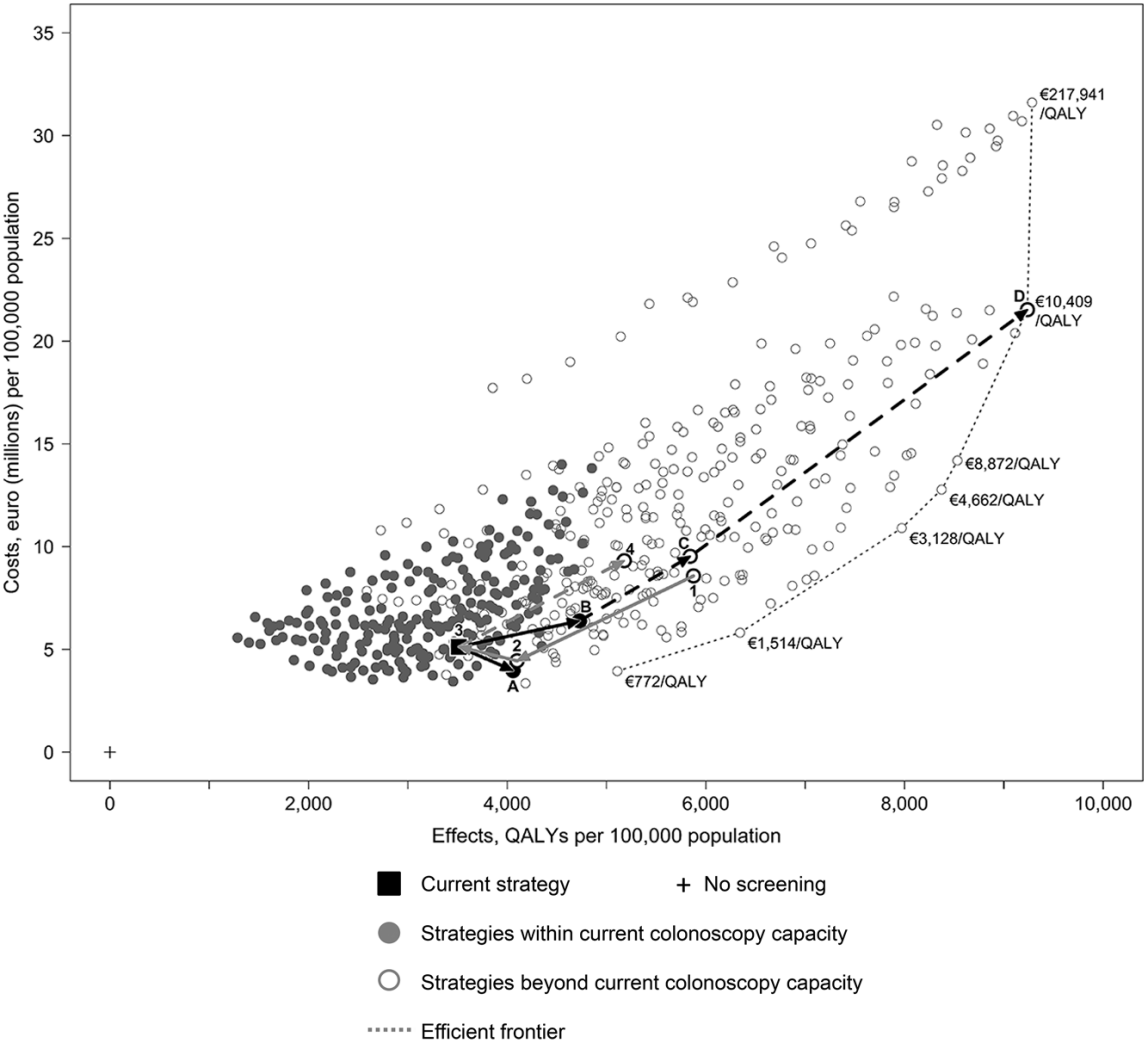
Past policy changes and future policy options including the optimal strategy without any colonoscopy capacity constraint at point D.

Finally, Figure 3 shows the overall optimally cost-effective strategy without any colonoscopy capacity constraint at point D, which uses annual screening between ages 50 to 80 y at a FIT cutoff of 10 µg Hb/g (50 ng Hb/mL). This would require a considerable increase in colonoscopy capacity of 691% relative to the status quo and would cost 320% more but would yield an estimated 163% more QALYs and 111% more CRC deaths prevented.

The optimally cost-effective strategy within the currently implied capacity constraint within each sensitivity analysis scenario is reported in Supplementary Appendix Table 2. While the sensitivity analysis finds that the optimally effective strategy within the capacity implied by the current policy varies between analyses, the general qualitative finding is that policies superior to the status quo can be found when broader age ranges and longer screening intervals are considered. However, it is notable that the most cost-effective strategies do not always feature the lowest FIT cutoff. Similarly, we find the same general result when applying alternative test performance characteristics to match a recent study presenting a similar analysis.

## Discussion

Our analysis shows that the optimal policy response to limited colonoscopy capacity may not be to raise the FIT cutoff level or widen the screening age range but rather to use longer screening intervals and more sensitive cutoff levels. In our case study, the current policy response to limited capacity has been to preserve biennial screening while narrowing the screening age range and raising the FIT cutoff. Modeling indicates that this runs counter to what makes the most effective use of scarce colonoscopy services. We find that by lengthening the screening interval, we can maintain a broad screening age range, retain a more sensitive FIT cutoff, and deliver greater benefits in terms of CRC deaths prevented. Costs would also be reduced by this approach. The primary explanation for our findings is the diminishing marginal returns of intensifying the frequency of screening: screening more people less often with a more sensitive FIT threshold seems a more efficient way of reducing the colonoscopy requirements than screening fewer people more frequently with a less sensitive FIT threshold.

Our findings are of clear policy relevance to the many countries facing difficulties in implementing CRC screening within constrained colonoscopy capacity, especially following the initial introduction of national programs.^45^ Restricting the screening age range and reducing the positivity threshold sensitivity of FIT appears a common policy response. A recent EU review of cancer screening services noted,

To optimise (limited) resource allocation, by maximising the cost-effectiveness ratio of the intervention, and to match their endoscopy capacity, several EU member states had actually adopted screening policies targeting a stricter age range, usually shifted to the older age groups, showing a higher prevalence of disease, resulting in a lower cost per lesion detected.^7^

Our results raise the possibility that these countries may also be making what seem like logical but potentially suboptimal policy responses to capacity constraints.

Our results differ from other studies of CRC screening using the same MISCAN-Colon model to estimate the optimal policy response to scarce colonoscopy capacity.^9,13,14^ The authors of those previous studies found that higher FIT cutoffs would be optimal under binding colonoscopy constraints. Our results differ from those of Van Hees et al., as, among other reasons, their analysis addressed a set of alternatives within an already limited screening age range as set out by policy makers.^9^ Similarly, our conclusions differ from those of Wilschut et al.^13^ and Goede et al.^14^ because we simulated a broader range of screening intervals, including every 4 and 5 y. As such, our analysis adds a novel finding to the literature on optimal CRC screening within constrained colonoscopy capacity.

A recently published analysis by Whyte et al.^36^ considering optimized screening in a UK context that considered variation in the age range, interval, and FIT cutoff provides a comparable analysis to our own. However, their conclusions differ, as they found that the optimal use of scarce capacity would be biennial screening with a 51- to 65-year age range and high FIT cutoff. This difference in conclusion may reflect a broader difference between the models, as Whyte et al. consistently found CRC screening to be net cost saving, whereas the MISCAN model found it to be net costly. Comparisons of FIT performance assumptions indicate that MISCAN assumes lower sensitivity for adenomas and higher sensitivity for CRC relative to Whyte et al. Although both models have been validated with trial data,^29,46^ the FIT performance for CRC as adopted in our MISCAN analyses is broadly concordant with meta-analysis estimates, and the data reported by Whyte et al provided advanced adenoma rates, which are significantly higher, and CRC rates, which are significantly lower.^47^ Even when applying the test performance characteristics as assumed by Whyte et al. in a sensitivity analysis, we still found that the optimal policy is in accordance with our general result and in contradiction to their findings. Further research may be required to resolve the reasons for the divergent conclusions.

Our findings illustrate the general principle that a cancer screening CEA should simulate a broad range of policy alternatives to find the optimal strategy. The initial HTA within the case study assessed only a small range of strategies and was published before work showing the benefit of varying FIT cutoffs. This led the analysis to overlook the issue of diminishing marginal returns of shortening the screening interval. Accordingly, the analysis could not identify the benefits of applying longer intervals to more people, rather than retaining short intervals for a narrow age range. Although the original HTA was supplemented by additional evaluations, these too did not consider strategies with longer intervals.^25^ A similar conclusion could therefore apply to many other European countries.

Within the specific context of Ireland, better CRC prevention will require careful planning. The Irish Cancer Society has raised concerns regarding colonoscopy capacity constraints and the emergence of inequities of access to colonoscopy between public and private patients.^48,49^ The recently renewed National Cancer Strategy restated the plan to expand capacity to permit the restoration of the screening age range to 55 to 74 y by the end of 2021.^27^ Plans for this ambitious capacity expansion are being managed by Ireland’s Health Service Executive National Endoscopy Steering Group.^27^

While the currently planned expansion of colonoscopy capacity is welcome, our results indicate that the case study program will remain unnecessarily inefficient. Modeling suggests that considerable improvements could be achieved if longer intervals of 4 y were adopted instead of the current 2-year interval. An increase in the screening interval could lead decision makers to worry that the public might become confused, and adherence could suffer. While such potential concerns are understandable, there is no evidence that adherence would be compromised, given the use of wider intervals in other disease areas. Conversely, the modeling evidence suggests that persisting with the present policy is likely to save fewer lives than other feasible strategies.

Our results also highlight a broader concern about the sufficiency of CRC screening programs in Ireland and other European nations. While Ireland plans to expand colonoscopy capacity, the current policy commitment still falls far short of what is ultimately required. Our results indicate that much larger gains could be made if annual screening were adopted while remaining cost-effective (strategy D, Figure 3). This again emphasizes the need for CEAs to consider a broad range of options. An overview of current screening policies in Europe is provided in Supplementary Appendix Table 1. The current predominance of biennial screening throughout Europe might lead policy makers to accept very considerable underprovision of CRC screening and save too few lives.

Our analysis naturally has some limitations. First, to date, no trial or observational data have examined the long-term effect of varying FIT intervals^50^; thus, the correlation between multiple tests and absolute risk, especially in the context of nonbleeding lesions, remains uncertain. Accordingly, the conclusions presented here on both extending the interval and using annual screening depend heavily on the current model assumptions. More data might be required to give decision makers confidence in varying the screening interval. Despite this, our analysis usefully illustrates what additional studies could be beneficial to undertake.

Second, the model reflects the incidence of disease and health care costs in the Netherlands and therefore can provide only a broad indication of what is likely to apply in an Irish context. Stage distribution patterns of CRC vary by time from screening implementation, coverage, and uptake.^51^ Before the implementation of screening, Ireland had a higher level of stage 4 and lower level of stage 1 disease than the Netherlands did. It is certainly possible that had we adapted the initial Dutch model configuration for Irish parameters, we might have found other policies to be optimal. Despite this, we believe this would be unlikely to alter our overall conclusion that consideration of a broader set of policy alternatives was likely to lead to better outcomes. Indeed, our results may underestimate the effectiveness of reconfigured screening programs considering the differences in prescreening implementation stage distribution patterns. The constraints facing Ireland are likely to be relevant for other European countries. By preserving the existing MISCAN model parameters and assessing only the relative policy differences applied and proposed in Ireland, we believe this provides a framework to highlight the principle of ensuring all relevant comparators are evaluated. Our work indicates it would be useful to establish whether the results presented here are still observed in an analysis fully adapted for an Irish context.

Furthermore, in common with many screening HTAs, we assumed 100% screening adherence. Currently, uptake within the national bowel cancer screening program is approximately 40%.^25^ Similarly, the FIT cutoff of 45 µg Hb/g as used in the program would generate fewer false positives than we inferred by using a 40-µg Hb/g cutoff. Consequently, our analysis may marginally overestimate current colonoscopy capacity. However, this approximation was necessary given the need for a consistent source for the test performance characteristics of the alternative FIT cutoffs. The model assumes 95% of colonoscopies reach the caecum. This may overestimate the effectiveness of the procedure, as studies have shown that this proportion can be lower.^52,53^ Finally, recent evidence has shown an increase in the incidence of CRC in European adults younger than 50 y.^54^ The model presented here does not represent such trends in CRC incidence and so may underestimate the potential benefits of policies that offer earlier start ages as a tradeoff against higher screening frequency. Despite these simplifications, we are confident that the analysis valuably illustrates the relevance of considering a broad range of policy alternatives and a clear indication of how a national bowel cancer screening program could save more lives.

An explicit acknowledgment of the relevance of the COVID-19 pandemic to our study is necessary. Our analysis was conceived before the advent of COVID-19 and does not reflect the additional capacity challenges that screening programs are now facing; however, the possibility that capacity constraints in CRC screening will be exacerbated in the medium term heightens the relevance of our conclusions.

Adopting annual FIT would require exceptionally large increases in colonoscopy capacity for many countries. In the Irish context, we suggest that a revision of the HTA evidence supporting the CRC screening program is now timely, both for the medium-term optimization of current capacity and the longer-term planning of overall colonoscopy capacity requirements. It is now necessary to revisit and expand previous analyses of CRC screening and consider additional policy alternatives. Such evidence and policy reviews are now required elsewhere in Europe too. Given that trials examining the effectiveness of FIT may not be available for another 10 years,^55^ modeling provides for more timely improvements. Given the interim shortfall in trial data, initiatives such as the EUTOPIA screening modeling project will be useful in assisting member states to inform such reviews.^56^

## Conclusion

Existing CRC screening programs may be unnecessarily ineffective and inefficient if analyses informing their design do not consider a wide range of strategies. In our case study, more lives and health services costs could be saved within existing colonoscopy capacity constraints if a lengthening of the screening interval was traded off against an increase in population coverage and the adoption of a more sensitive FIT cutoff. A broader finding is that much larger increases in CRC screening capacity than is currently planned appear warranted if annual screening were to be adopted. Policy makers must recognize the need to consider all policy alternatives, within both current colonoscopy capacity constraints and future expanded service capacity. Otherwise, many avoidable CRC deaths will result over the coming decades. The findings from this case study are likely to be highly relevant for all European nations implementing FIT-based CRC screening with biennial intervals in the face of constrained colonoscopy capacity.

## Supplemental Material

sj-docx-1-mpp-10.1177_23814683221097064 – Supplemental material for Colorectal Cancer Screening within Colonoscopy Capacity Constraints: Can FIT-Based Programs Save More Lives by Trading off More Sensitive Test Cutoffs against Longer Screening Intervals? Click here for additional data file. Supplemental material, sj-docx-1-mpp-10.1177_23814683221097064 for Colorectal Cancer Screening within Colonoscopy Capacity Constraints: Can FIT-Based Programs Save More Lives by Trading off More Sensitive Test Cutoffs against Longer Screening Intervals? by Ethna McFerran, James F. O’Mahony, Steffie Naber, Linda Sharp, Ann G. Zauber, Iris Lansdorp-Vogelaar and Frank Kee in MDM Policy & Practice
