# Supplementary material for: Colorectal Cancer Screening within Colonoscopy Capacity Constraints: Can FIT-Based Programs Save More Lives by Trading off More Sensitive Test Cutoffs against Longer Screening Intervals?
Source: MDM Policy Pract. 2022 May 7;7(1):23814683221097064. doi: 10.1177/23814683221097064 (PMC9091364; doi:10.1177/23814683221097064)
Supplement: sj-docx-1-mpp-10.1177_23814683221097064 – Supplemental material for Colorectal Cancer Screening within Colonoscopy Capacity Constraints: Can FIT-Based Programs Save More Lives by Trading off More Sensitive Test Cutoffs against Longer Screening Intervals? [file sj-docx-1-mpp-10.1177_23814683221097064.docx]

**Appendices**

| Country | Programme initiation | Target age range | Screening interval in years  (Screening modality) | FIT cut-off  (Where available,  converted to µgHb/g) |
| --- | --- | --- | --- | --- |
| Austria¹ | 2003 | 40+gFOBT; 50+ Colo¹ | 1 (FIT); 7-10 (Colo)¹ | - |
|  |  | 40-80 (Bergenland) | 1 (gFOBT) |  |
| Belgium | 2009² | 50-74 (Wallonia/ Brussels) | 2 FIT | 15μg Hb/g  (FIT as of 2016) |
|  | 2013 | 56-74 (Flanders) | 2 (FIT); 10 (Colo) | 15μg Hb/g |
| Bulgaria |  | - | - |  |
| Croatia | 2008 | 50-74 | 2 (gFOBT) | - |
| Cyprus | 2013 | 50-69 | 2 (gFOBT) | - |
| Czech Republic | 2000³ | 50/ 55+ ^3^ | 1 (FIT;50-54);2 (FIT;55+)  10 (Colo) | 15μg Hb/g |
| Denmark | 2014 | 50-74 | 2 (FIT) | 20 μg Hb/g |
| Estonia⁴ | 2016⁴ | 60-69⁴ | 2 (FIT) | 20 μg Hb/g |
| Finland | 2004 | 60-69 | 2 (FIT) | FIT as of 2019  25 μg Hb/g for Females  70 μg Hb/g for Males |
| France^14^ | 2002 | 50-74 | 2 (FIT) | 30 µg Hb/g |
| Germany⁵ | 1974⁵ | 50-74 | 1 (50-54 gFOBT); 2 (55+ gFOBT)  10⁵ (55+ Colo) | Conversion to FIT underway (2017) |
| Greece | - | 50-70 | 2 (gFOBT); 5 (Colo) | - |
| Hungary | 2007 | 50-70 | 2 (gFOBT) | - |
| Ireland | 2012 | 60-69⁷ | 2 (FIT) | 40 μg Hb/g ^25^ |
| Italy | 1982⁸ | 50-69 | 2 (FIT) | 20 μg Hb/g |
| Latvia | 2009 | 50-74 | 1 (gFOBT) | - |
| Lithuania | 2009⁶ | 50-74 | 2 (FIT) | 6-40 μg Hb/g |
| Luxembourg¹⁰ | 2016 | 55-74 | 2 (FIT) | 50 μg Hb/g |
| Malta | 2013 | 55-66 | 2 (FIT) | 20 μg Hb/g |
| Netherlands | 2014 | 55-75 | 2 (FIT) | 47 μg Hb/g ^19^ |
| Norway | - | - | - | - |
| Poland | 2012 | 55-64 | 10+ | - |
| Portugal | 2017 | 50-75 (Algarve) | 2 (FIT) | 117 μg Hb/g (preliminary data) |
|  | 2018 | 50-74 |  | 20 μg Hb/g |
| Romania | - | - | - | - |
| Slovak Republic | - | - | - | - |
| Slovenia | 2009 | 50-74 | 2 (FIT) | 67 μg Hb/g |
| Spain | 2000 | 50-69 | 2 (gFOBT) | - |
|  |  | 50-69 (Basque Country) | 2 (FIT) | 20 μg Hb/g |
| Sweden¹¹ | 2008 | 60-69 | 2 (FIT) | 40 and 80 µg Hb/g for women and men, respectively |
| United Kingdom | 2006¹² | 60-74 (England)  *2018 recommendation to lower start age to 50* | 2 gFOBT/ FIT (rolling introduction) | England pilot sites 180 µg Hb/g, 120 µg Hb/g in some areas |
|  |  | 50-74 (Scotland) | 2 FIT | Scotland 80 ug Hb/g since Nov 2017 |
|  |  | 60-74 (Northern Ireland) | 2 gFOBT/ FIT (planned introduction) | FIT Introduction planned 2021 |
| Switzerland^13^ | 2015 | 50-59 (*Canton of Vaud) | 2 (FIT); 10 (Colo) | 15 µg Hb/g |

**Appendix Table 1** Screening programmes across European countries

**¹Austria** - population-based FIT screening programme implemented only in Burgenland (2003), in the rest of the country, opportunistic screening where an annual check, a gFOBT is offered, the colonoscopy interval is 10 years. ²**Belgium** - In March 2016 Wallonia-Brussels replaced gFOBT with FIT. **^3^**The population-based programme in the **Czech Republic** started in 2014. ⁴In **Estonia,** the population-based pilot programme started in 2016, intended target group of 60-69 years old; ⁵In **Germany**, opportunistic gFOBT screening activities started in 1974, a population-based programme started 2019. Colonoscopy continues, recommended twice for those older than 55 years. ⁶In **Hungary**, women who have already been screened opportunistically were not invited; ⁷**Ireland** committed to planned age extension to target age to 55-74-year-olds; ⁸In **Italy**, screening started in 1982 in Florence, and between 2000 and 2004 in other regions. The majority of programmes employ FIT (apart from Piedmont region which adopted Flexible Sigmoidoscopy and FIT for non-responders) for subjects aged from 50 to 69 or 70 years; ⁹In **Lithuania**, population-based programme started in 2009 in two districts, decentralised national testing available from 2014, with a pilot with two centralised labs due to commence 2021. ¹⁰In **Luxembourg** a population-based programme started in 2016; ¹¹In **Sweden,** only Stockholm Gotland region introduced screening in 2008, and then switched to FIT in 2015 ^57^; ¹²Year of programme initiation: gFOBT **England** 2006, gFOBT **Northern** Ireland 2010, gFOBT **Scotland** 2007, gFOBT **Wales** 2008, as of 2016 the UK Screening Committee recommended the adoption of FIT, rolling introduction ^17,58^ with Northern Ireland the last region to adopt in late 2021. ^13^In **Switzerland**, A 2013 law authorized reimbursement by basic health insurance of either FIT every 2 years or colonoscopy every 10 years for adults aged 50 to 69 years. In 2015, the Canton of Vaud launched the first large-scale, organized CRC screening program in Switzerland. ^14^ In **France,** FIT screening replaced gFOBT with FIT in April 2015.

**References**

57. Blom J, Lo¨ wbeer C, Elfstro¨m KM, et al. Gender-specific cut-offs in colorectal cancer screening with FIT: increased compliance and equal positivity rate. *J Med Screen* 2018;26(2):92–7. doi:10.1177/0969141318804843

58. Mackie A, Halloran S. Moving from guaiac faecal occult blood test (gFOBT) to a faecal immunochemical test for haemoglobin (FIT) in the bowel screening programme: a consultation. 2015. Available from: <http://legacy.scree>ning.nhs.uk/bowelcancer. Accessed December 28, 2017

**Appendix Table 2** Sensitivity analysis of the optimally cost-effective policy under currently implied capacity constraints

| **Scenario** | **Low** | | | **High** | | |
| --- | --- | --- | --- | --- | --- | --- |
| Incidence | 50% below base case | | | 50% above base case | | |
| Optimal policy | Age range | Interval, years | FIT Cut-off | Age range | Interval, years | FIT Cut-off |
|  | 45-77 | 4 | 30 | 55-80 | 5 | 10 |
| FIT screening participation | 50% | | | 80% | | |
| Optimal policy | Age range | Interval, years | FIT Cut-off | Age range | Interval, years | FIT Cut-off |
|  | 50-80 | 5 | 15 | 55-75 | 5 | 10 |
| Discount rate | 1.5% | | | 5.0% | | |
| Optimal policy | Age range | Interval, years | FIT Cut-off | Age range | Interval, years | FIT Cut-off |
|  | 55-75 | 5 | 10 | 45-81 | 4 | 30 |
| Test performance | Matching Whyte et al.’s assumptions | | | | | |
| Optimal policy | Age range | | Interval, years | | FIT Cut-off | |
|  | 55-79 | | 4 | | 20 | |
| All FIT cut-offs expressed as µg Hb/g | | | | | | |

**APPENDIX Table 3** Results of All Strategies Modelled (sorted by FIT cut-off)

| **Strategy Characteristics** | | | | | | **Estimated Outcomes (for 10 million persons simulated)** | | | | **Comparisons Relative to Current Policy** | | | | **Total CRC deaths prevented** | **ICER, €/QALY** |
| --- | --- | --- | --- | --- | --- | --- | --- | --- | --- | --- | --- | --- | --- | --- | --- |
| **Policy Position** | **Cut off (μg Hb/g)** | **Interval (Years)** | **Start Age** | **Stop Age** | **Number of Lifetime Screens** | **∆QALY** | **∆C (€)** | **∆LYG** | **Total Number of Colonoscopies** | **Within Capacity 0= No**  **1= Yes** | **% ∆QALY** | **% ∆C** | **%∆No. Colonoscopies** |  |  |
| No Screening | - | - | - | - | - | - | - | - | - | - | - | - | - | - | - |
| Max overall Net Health Benefit (D) | 10 | 1 | 50 | 80 | 30 | 923,774 | 2,152,841,980 | 962,802 | 36,690,721 | 0 | 163 | 320 | 691 | 91186 | 10409 |
|  | 10 | 1 | 50 | 75 | 25 | 911,291 | 2,038,845,024 | 948,937 | 36,029,947 | 0 | 159 | 298 | 677 | 88557 | - |
|  | 10 | 1 | 50 | 70 | 20 | 878,990 | 1,889,923,187 | 914,974 | 34,438,993 | 0 | 150 | 269 | 643 | 83408 | - |
|  | 10 | 1 | 55 | 80 | 25 | 853,279 | 1,419,041,116 | 892,270 | 32,412,179 | 0 | 143 | 177 | 599 | 90489 | 8872 |
|  | 10 | 1 | 55 | 75 | 20 | 837,341 | 1,277,636,957 | 874,613 | 31,587,240 | 0 | 138 | 150 | 581 | 87173 | 4662 |
|  | 10 | 1 | 45 | 80 | 35 | 928,401 | 3,161,158,158 | 969,447 | 38,998,864 | 0 | 164 | 517 | 741 | 87349 | 217941 |
|  | 10 | 1 | 45 | 75 | 30 | 918,185 | 3,070,154,354 | 958,158 | 38,465,302 | 0 | 161 | 500 | 729 | 85224 | - |
|  | 10 | 1 | 45 | 70 | 25 | 892,149 | 2,948,308,364 | 930,661 | 37,189,552 | 0 | 154 | 476 | 702 | 81081 | - |
|  | 10 | 1 | 55 | 70 | 15 | 797,157 | 1,090,283,748 | 832,305 | 29,563,393 | 0 | 127 | 113 | 538 | 80744 | 3128 |
|  | 10 | 1 | 50 | 65 | 15 | 811,243 | 1,695,206,231 | 845,463 | 31,394,594 | 0 | 131 | 231 | 577 | 74679 | - |
|  | 10 | 1 | 45 | 65 | 20 | 837,582 | 2,792,411,142 | 874,754 | 34,789,084 | 0 | 138 | 445 | 650 | 74040 | - |
|  | 10 | 1 | 55 | 65 | 10 | 709,224 | 857,943,179 | 742,492 | 25,578,155 | 0 | 102 | 68 | 452 | 69389 | - |
|  | 10 | 1 | 60 | 80 | 20 | 706,852 | 986,260,798 | 749,691 | 26,540,438 | 0 | 101 | 93 | 472 | 83750 | - |
|  | 10 | 1 | 60 | 75 | 15 | 686,998 | 809,446,643 | 727,648 | 25,490,509 | 0 | 96 | 58 | 450 | 79597 | - |
|  | 10 | 1 | 50 | 60 | 10 | 685,214 | 1,423,396,387 | 718,065 | 26,200,406 | 0 | 95 | 178 | 465 | 61184 | - |
|  | 10 | 1 | 45 | 60 | 15 | 740,870 | 2,563,015,440 | 776,495 | 30,771,718 | 0 | 111 | 401 | 564 | 63639 | - |
|  | 10 | 1 | 60 | 70 | 10 | 634,347 | 581,066,953 | 672,429 | 22,818,047 | 0 | 81 | 13 | 392 | 71121 | 1514 |
|  | 10 | 1 | 65 | 80 | 15 | 523,101 | 791,176,426 | 570,808 | 19,654,061 | 0 | 49 | 55 | 324 | 72051 | - |
|  | 10 | 1 | 45 | 55 | 10 | 586,770 | 2,190,946,536 | 620,525 | 24,617,684 | 0 | 67 | 328 | 431 | 49193 | - |
|  | 10 | 1 | 65 | 75 | 10 | 496,510 | 571,947,109 | 541,520 | 18,263,474 | 0 | 41 | 12 | 294 | 66537 | - |
|  | 10 | 1 | 70 | 80 | 10 | 334,539 | 738,080,543 | 385,218 | 12,362,713 | 0 | -5 | 44 | 167 | 56490 | - |
|  | 10 | 2 | 50 | 80 | 15 | 802,314 | 1,444,752,482 | 872,082 | 17,539,998 | 0 | 128 | 182 | 278 | 84887 | - |
|  | 10 | 2 | 45 | 81 | 18 | 828,387 | 2,123,170,365 | 900,496 | 19,368,532 | 0 | 136 | 315 | 318 | 84566 | - |
|  | 10 | 2 | 50 | 76 | 13 | 789,494 | 1,345,952,060 | 855,800 | 17,124,596 | 0 | 125 | 163 | 269 | 81704 | - |
|  | 10 | 2 | 45 | 75 | 15 | 810,525 | 1,992,527,265 | 878,106 | 18,804,614 | 0 | 131 | 289 | 306 | 80251 | - |
|  | 10 | 2 | 45 | 71 | 13 | 782,377 | 1,901,606,608 | 845,891 | 18,089,982 | 0 | 123 | 271 | 290 | 75224 | - |
|  | 10 | 2 | 50 | 70 | 10 | 741,534 | 1,188,515,223 | 800,689 | 15,895,718 | 0 | 111 | 132 | 243 | 73277 | - |
|  | 10 | 2 | 55 | 81 | 13 | 723,695 | 1,003,088,247 | 792,025 | 15,090,209 | 0 | 106 | 96 | 225 | 82307 | - |
|  | 10 | 2 | 55 | 75 | 10 | 701,455 | 839,544,597 | 764,056 | 14,372,371 | 0 | 100 | 64 | 210 | 76944 | - |
|  | 10 | 2 | 55 | 71 | 8 | 665,232 | 723,266,647 | 722,585 | 13,441,645 | 0 | 89 | 41 | 190 | 70506 | - |
|  | 10 | 2 | 50 | 66 | 8 | 682,599 | 1,086,265,340 | 736,108 | 14,526,217 | 0 | 94 | 112 | 213 | 65015 | - |
|  | 10 | 2 | 45 | 65 | 10 | 702,995 | 1,762,301,445 | 759,134 | 16,296,739 | 0 | 100 | 244 | 251 | 64317 | - |
|  | 10 | 2 | 60 | 80 | 10 | 592,420 | 705,902,275 | 658,520 | 12,035,801 | 0 | 69 | 38 | 160 | 74578 | - |
|  | 10 | 2 | 60 | 76 | 8 | 575,371 | 583,723,782 | 637,209 | 11,493,660 | 0 | 64 | 14 | 148 | 70415 | - |
|  | 10 | 2 | 45 | 61 | 8 | 619,463 | 1,652,385,064 | 670,127 | 14,509,698 | 0 | 76 | 223 | 213 | 54906 | - |
|  | 10 | 2 | 55 | 65 | 5 | 560,257 | 558,610,770 | 608,155 | 11,004,228 | 0 | 59 | 9 | 137 | 55976 | - |
|  | 10 | 2 | 60 | 70 | 5 | 510,850 | 394,125,332 | 563,686 | 9,804,082 | 0 | 45 | -23 | 111 | 59017 | 772 |
|  | 10 | 2 | 50 | 60 | 5 | 535,459 | 909,694,154 | 579,400 | 11,372,549 | 0 | 52 | 78 | 145 | 48455 | - |
|  | 10 | 2 | 65 | 81 | 8 | 438,231 | 629,411,196 | 502,881 | 8,984,357 | 0 | 25 | 23 | 94 | 64417 | - |
|  | 10 | 2 | 65 | 75 | 5 | 406,673 | 425,304,283 | 464,060 | 7,979,930 | 0 | 16 | -17 | 72 | 56887 | - |
|  | 10 | 2 | 45 | 55 | 5 | 446,112 | 1,393,734,864 | 486,162 | 10,823,546 | 0 | 27 | 172 | 133 | 37968 | - |
|  | 10 | 2 | 70 | 80 | 5 | 275,209 | 603,227,178 | 334,639 | 5,650,250 | 0* | -22 | 18 | 22 | 49100 | - |
|  | 10 | 3 | 45 | 81 | 12 | 723,132 | 1,725,591,172 | 807,480 | 11,959,541 | 0 | 106 | 237 | 158 | 77229 | - |
|  | 10 | 3 | 50 | 80 | 10 | 691,806 | 1,219,123,010 | 774,726 | 10,834,242 | 0 | 97 | 138 | 134 | 76616 | - |
|  | 10 | 3 | 45 | 75 | 10 | 704,712 | 1,586,774,580 | 782,403 | 11,479,280 | 0 | 101 | 210 | 148 | 72326 | - |
|  | 10 | 3 | 50 | 74 | 8 | 667,403 | 1,068,268,766 | 742,491 | 10,245,532 | 0 | 90 | 109 | 121 | 70565 | - |
|  | 10 | 3 | 50 | 71 | 7 | 642,359 | 995,380,953 | 712,249 | 9,780,945 | 0 | 83 | 94 | 111 | 65894 | - |
|  | 10 | 3 | 45 | 69 | 8 | 655,535 | 1,452,838,258 | 723,613 | 10,624,442 | 0 | 87 | 184 | 129 | 63539 | - |
|  | 10 | 3 | 55 | 79 | 8 | 618,771 | 833,750,463 | 696,773 | 9,201,050 | 0 | 76 | 63 | 98 | 72665 | - |
|  | 10 | 3 | 55 | 76 | 7 | 607,300 | 750,310,851 | 681,183 | 8,901,165 | 0 | 73 | 47 | 92 | 69630 | - |
|  | 10 | 3 | 45 | 66 | 7 | 614,689 | 1,394,869,637 | 677,239 | 10,004,383 | 0 | 75 | 172 | 116 | 57774 | - |
|  | 10 | 3 | 55 | 70 | 5 | 555,147 | 592,314,553 | 618,020 | 7,901,249 | 0 | 58 | 16 | 70 | 59730 | - |
|  | 10 | 3 | 50 | 65 | 5 | 557,050 | 865,773,932 | 614,793 | 8,406,382 | 0 | 59 | 69 | 81 | 53501 | - |
|  | 10 | 3 | 60 | 81 | 7 | 511,883 | 672,359,371 | 587,074 | 7,349,506 | 0* | 46 | 31 | 58 | 67378 | - |
|  | 10 | 3 | 60 | 75 | 5 | 487,855 | 496,380,554 | 554,500 | 6,712,099 | 0* | 39 | -3 | 45 | 60979 | - |
|  | 10 | 3 | 45 | 60 | 5 | 495,488 | 1,271,389,179 | 546,082 | 8,306,459 | 0 | 41 | 148 | 79 | 43868 | - |
|  | 10 | 3 | 55 | 64 | 3 | 448,274 | 462,275,645 | 497,758 | 6,212,031 | 0* | 28 | -10 | 34 | 44965 | - |
|  | 10 | 3 | 60 | 69 | 3 | 418,359 | 335,033,755 | 472,381 | 5,500,475 | 0* | 19 | -35 | 19 | 48630 | - |
|  | 10 | 3 | 50 | 59 | 3 | 414,833 | 728,871,279 | 459,007 | 6,349,948 | 0* | 18 | 42 | 37 | 37485 | - |
|  | 10 | 3 | 65 | 80 | 5 | 373,947 | 569,414,230 | 443,045 | 5,496,182 | 0* | 6 | 11 | 19 | 56626 | - |
|  | 10 | 3 | 65 | 74 | 3 | 338,594 | 376,439,372 | 397,295 | 4,657,950 | 0* | -4 | -26 | 0 | 48047 | - |
|  | 10 | 3 | 45 | 54 | 3 | 336,341 | 1,064,714,167 | 373,269 | 5,983,644 | 0* | -4 | 108 | 29 | 28372 | - |
|  | 10 | 3 | 70 | 79 | 3 | 233,446 | 537,708,524 | 293,457 | 3,647,597 | 1* | -34 | 5 | -21 | 42708 | - |
|  | 10 | 4 | 45 | 81 | 9 | 634,650 | 1,531,006,698 | 723,683 | 8,436,251 | 0 | 81 | 199 | 82 | 70096 | - |
|  | 10 | 4 | 45 | 77 | 8 | 626,646 | 1,434,333,688 | 710,842 | 8,176,163 | 0 | 78 | 180 | 76 | 67303 | - |
|  | 10 | 4 | 50 | 82 | 8 | 608,340 | 1,151,702,200 | 696,295 | 7,668,753 | 0 | 73 | 125 | 65 | 70213 | - |
| Optimised (Maximum  Net Health Benefit)  with expanded  capacity (C) | 10 | 4 | 50 | 74 | 6 | 584,285 | 952,705,745 | 661,351 | 7,065,385 | 0* | 66 | 86 | 52 | 63306 | - |
|  | 10 | 4 | 45 | 69 | 6 | 572,330 | 1,253,621,886 | 642,319 | 7,358,045 | 0 | 63 | 145 | 59 | 56515 | - |
|  | 10 | 4 | 50 | 70 | 5 | 551,155 | 857,413,543 | 619,985 | 6,583,323 | 0* | 57 | 67 | 42 | 56997 | - |
|  | 10 | 4 | 55 | 79 | 6 | 544,582 | 776,942,952 | 625,176 | 6,502,716 | 0* | 55 | 52 | 40 | 65848 | - |
|  | 10 | 4 | 55 | 75 | 5 | 528,949 | 668,137,370 | 603,165 | 6,145,744 | 0* | 51 | 30 | 33 | 61562 | - |
|  | 10 | 4 | 55 | 71 | 4 | 496,870 | 565,688,439 | 562,802 | 5,638,202 | 0* | 41 | 10 | 22 | 55137 | - |
|  | 10 | 4 | 45 | 65 | 5 | 519,871 | 1,181,088,410 | 581,174 | 6,735,302 | 0* | 48 | 131 | 45 | 48901 | - |
|  | 10 | 4 | 50 | 66 | 4 | 499,193 | 777,776,913 | 558,903 | 5,939,650 | 0* | 42 | 52 | 28 | 49088 | - |
|  | 10 | 4 | 60 | 80 | 5 | 463,505 | 673,509,708 | 540,643 | 5,576,630 | 0* | 32 | 32 | 20 | 63020 | - |
|  | 10 | 4 | 55 | 67 | 3 | 446,037 | 480,511,662 | 502,330 | 4,955,736 | 0* | 27 | -6 | 7 | 46886 | - |
|  | 10 | 4 | 60 | 76 | 4 | 436,522 | 507,372,483 | 506,403 | 4,870,522 | 0* | 24 | -1 | 5 | 56323 | - |
|  | 10 | 4 | 45 | 61 | 4 | 448,757 | 1,108,387,476 | 500,926 | 5,947,920 | 0* | 28 | 116 | 28 | 40349 | - |
|  | 10 | 4 | 50 | 62 | 3 | 426,384 | 706,498,688 | 476,562 | 5,116,824 | 0* | 21 | 38 | 10 | 39950 | - |
| Maximum Net Health Benefit with  cost saving (A) | 10 | 4 | 60 | 72 | 3 | 405,847 | 396,797,580 | 467,287 | 4,368,403 | 1* | 16 | -23 | -6 | 49801 | - |
|  | 10 | 4 | 45 | 57 | 3 | 360,844 | 1,016,977,789 | 403,308 | 4,981,988 | 0* | 3 | 99 | 7 | 31139 | - |
|  | 10 | 4 | 65 | 81 | 4 | 332,405 | 587,973,994 | 403,421 | 3,962,115 | 1* | -5 | 15 | -15 | 52048 | - |
|  | 10 | 4 | 65 | 77 | 3 | 320,785 | 463,363,110 | 385,414 | 3,600,857 | 1* | -9 | -10 | -22 | 48142 | - |
|  | 10 | 4 | 70 | 82 | 3 | 214,632 | 620,130,770 | 278,324 | 2,922,624 | 1* | -39 | 21 | -37 | 41424 | - |
|  | 10 | 5 | 45 | 80 | 7 | 567,502 | 1,377,043,855 | 655,746 | 6,461,558 | 0* | 62 | 169 | 39 | 63939 | - |
|  | 10 | 5 | 45 | 75 | 6 | 554,118 | 1,254,714,856 | 635,199 | 6,148,904 | 0* | 58 | 145 | 33 | 59754 | - |
|  | 10 | 5 | 50 | 80 | 6 | 540,902 | 1,032,069,961 | 626,544 | 5,752,420 | 0* | 54 | 102 | 24 | 63240 | - |
|  | 10 | 5 | 50 | 75 | 5 | 526,696 | 904,318,681 | 604,872 | 5,421,222 | 0* | 50 | 77 | 17 | 58823 | - |
|  | 10 | 5 | 45 | 70 | 5 | 520,726 | 1,142,045,189 | 592,025 | 5,703,863 | 0* | 48 | 123 | 23 | 52903 | - |
|  | 10 | 5 | 50 | 70 | 4 | 490,702 | 786,836,047 | 558,424 | 4,950,911 | 0* | 40 | 54 | 7 | 51481 | - |
|  | 10 | 5 | 55 | 80 | 5 | 487,965 | 773,449,220 | 569,219 | 4,900,614 | 0* | 39 | 51 | 6 | 60535 | - |
| Maximum Net Health Benefit  within capacity (B) | 10 | 5 | 55 | 75 | 4 | 472,789 | 638,614,456 | 546,134 | 4,549,020 | 1* | 35 | 25 | -2 | 55821 | - |
|  | 10 | 5 | 45 | 65 | 4 | 463,521 | 1,052,653,478 | 523,459 | 5,110,920 | 0* | 32 | 106 | 10 | 44059 | - |
|  | 10 | 5 | 55 | 70 | 3 | 434,759 | 515,284,785 | 497,073 | 4,045,291 | 1* | 24 | 1 | -13 | 48018 | - |
|  | 10 | 5 | 50 | 65 | 3 | 430,174 | 692,863,637 | 485,786 | 4,316,919 | 1* | 22 | 35 | -7 | 42091 | - |
|  | 10 | 5 | 60 | 80 | 4 | 403,866 | 618,230,470 | 479,923 | 3,991,031 | 1* | 15 | 21 | -14 | 55257 | - |
|  | 10 | 5 | 60 | 75 | 3 | 387,639 | 475,577,032 | 455,246 | 3,611,471 | 1* | 10 | -7 | -22 | 50212 | - |
|  | 10 | 5 | 55 | 65 | 2 | 369,220 | 419,017,019 | 418,820 | 3,355,957 | 1* | 5 | -18 | -28 | 37875 | - |
|  | 10 | 5 | 45 | 60 | 3 | 379,983 | 974,232,295 | 428,176 | 4,348,822 | 1* | 8 | 90 | -6 | 33938 | - |
|  | 10 | 5 | 60 | 70 | 2 | 345,527 | 343,881,706 | 401,089 | 3,055,984 | 1* | -2 | -33 | -34 | 41599 | - |
|  | 10 | 5 | 50 | 60 | 2 | 340,757 | 610,099,492 | 383,614 | 3,492,848 | 1* | -3 | 19 | -25 | 31208 | - |
|  | 10 | 5 | 65 | 80 | 3 | 298,932 | 551,237,692 | 367,609 | 3,067,574 | 1* | -15 | 8 | -34 | 47215 | - |
|  | 10 | 5 | 65 | 75 | 2 | 280,903 | 395,944,154 | 340,307 | 2,649,399 | 1* | -20 | -23 | -43 | 41626 | - |
|  | 10 | 5 | 45 | 55 | 2 | 277,658 | 860,510,920 | 313,313 | 3,400,608 | 1* | -21 | 68 | -27 | 23507 | - |
|  | 10 | 5 | 70 | 80 | 2 | 193,019 | 539,979,484 | 252,102 | 2,185,244 | 1* | -45 | 5 | -53 | 36944 | - |
|  | 15 | 1 | 50 | 80 | 30 | 885,609 | 2,150,147,793 | 953,719 | 33,945,738 | 0 | 152 | 320 | 632 | 91214 | - |
|  | 15 | 1 | 50 | 75 | 25 | 867,906 | 2,008,183,578 | 933,175 | 33,209,233 | 0 | 147 | 292 | 616 | 87381 | - |
|  | 15 | 1 | 45 | 80 | 35 | 909,275 | 3,096,192,914 | 981,570 | 36,997,989 | 0 | 159 | 505 | 698 | 89670 | - |
|  | 15 | 1 | 45 | 75 | 30 | 893,801 | 2,975,861,303 | 963,697 | 36,367,643 | 0 | 154 | 481 | 684 | 86358 | - |
|  | 15 | 1 | 55 | 80 | 25 | 806,697 | 1,454,368,293 | 871,536 | 29,286,716 | 0 | 130 | 184 | 532 | 88908 | - |
|  | 15 | 1 | 50 | 70 | 20 | 825,582 | 1,839,383,141 | 886,891 | 31,474,667 | 0 | 135 | 259 | 579 | 80478 | - |
|  | 15 | 1 | 55 | 75 | 20 | 785,530 | 1,290,065,772 | 847,062 | 28,422,491 | 0 | 124 | 152 | 513 | 84364 | - |
|  | 15 | 1 | 45 | 70 | 25 | 858,183 | 2,828,337,696 | 924,693 | 34,884,576 | 0 | 144 | 452 | 652 | 80529 | - |
|  | 15 | 1 | 55 | 70 | 15 | 735,743 | 1,092,206,037 | 792,666 | 26,337,881 | 0 | 109 | 113 | 468 | 76246 | - |
|  | 15 | 1 | 50 | 65 | 15 | 744,930 | 1,635,877,295 | 801,275 | 28,269,083 | 0 | 112 | 219 | 510 | 69977 | - |
|  | 15 | 1 | 45 | 65 | 20 | 789,294 | 2,652,234,312 | 851,586 | 32,203,284 | 0 | 125 | 418 | 594 | 71565 | - |
|  | 15 | 1 | 60 | 80 | 20 | 661,581 | 1,030,873,996 | 725,098 | 23,405,975 | 0 | 88 | 101 | 405 | 81205 | - |
|  | 15 | 1 | 60 | 75 | 15 | 636,347 | 838,312,463 | 695,923 | 22,365,533 | 0 | 81 | 64 | 382 | 75775 | - |
|  | 15 | 1 | 55 | 65 | 10 | 636,977 | 863,045,712 | 688,211 | 22,370,084 | 0 | 81 | 69 | 382 | 63363 | - |
|  | 15 | 1 | 45 | 60 | 15 | 676,617 | 2,406,521,360 | 733,203 | 27,929,697 | 0 | 93 | 370 | 502 | 59346 | - |
|  | 15 | 1 | 60 | 70 | 10 | 575,369 | 611,537,554 | 629,627 | 19,751,682 | 0 | 64 | 19 | 326 | 65789 | - |
|  | 15 | 1 | 50 | 60 | 10 | 608,386 | 1,362,001,589 | 658,568 | 23,056,080 | 0 | 73 | 166 | 397 | 55258 | - |
|  | 15 | 1 | 65 | 80 | 15 | 485,781 | 819,627,956 | 547,921 | 16,859,864 | 0 | 38 | 60 | 264 | 69125 | - |
|  | 15 | 1 | 65 | 75 | 10 | 454,262 | 594,450,072 | 511,730 | 15,551,819 | 0 | 29 | 16 | 235 | 62400 | - |
|  | 15 | 1 | 45 | 55 | 10 | 514,212 | 2,022,129,051 | 562,876 | 21,745,446 | 0 | 46 | 295 | 369 | 43977 | - |
|  | 15 | 1 | 70 | 80 | 10 | 308,442 | 738,697,957 | 367,287 | 10,255,080 | 0 | -12 | 44 | 121 | 53668 | - |
|  | 15 | 2 | 45 | 81 | 18 | 769,895 | 2,057,488,235 | 866,988 | 16,469,886 | 0 | 119 | 302 | 255 | 82096 | - |
|  | 15 | 2 | 50 | 80 | 15 | 735,548 | 1,444,113,899 | 828,418 | 14,659,237 | 0 | 109 | 182 | 216 | 80981 | - |
|  | 15 | 2 | 50 | 76 | 13 | 720,404 | 1,331,638,049 | 808,151 | 14,272,441 | 0 | 105 | 160 | 208 | 77073 | - |
|  | 15 | 2 | 45 | 75 | 15 | 748,095 | 1,906,034,325 | 838,300 | 15,923,283 | 0 | 113 | 272 | 243 | 76607 | - |
|  | 15 | 2 | 45 | 71 | 13 | 714,781 | 1,805,924,149 | 798,669 | 15,240,898 | 0 | 103 | 253 | 229 | 70577 | - |
|  | 15 | 2 | 50 | 70 | 10 | 666,327 | 1,162,201,939 | 743,307 | 13,144,397 | 0 | 90 | 127 | 183 | 67312 | - |
|  | 15 | 2 | 55 | 81 | 13 | 659,853 | 1,041,042,150 | 748,216 | 12,485,887 | 0 | 88 | 103 | 169 | 78057 | - |
|  | 15 | 2 | 55 | 75 | 10 | 634,338 | 861,730,404 | 714,472 | 11,826,790 | 0 | 81 | 68 | 155 | 71631 | - |
|  | 15 | 2 | 55 | 71 | 8 | 594,817 | 741,262,291 | 667,374 | 10,988,441 | 0 | 69 | 45 | 137 | 64400 | - |
|  | 15 | 2 | 50 | 66 | 8 | 604,039 | 1,056,495,208 | 672,376 | 11,907,407 | 0 | 72 | 106 | 157 | 58434 | - |
|  | 15 | 2 | 45 | 65 | 10 | 628,902 | 1,653,603,954 | 700,933 | 13,568,232 | 0 | 79 | 223 | 193 | 58487 | - |
|  | 15 | 2 | 60 | 80 | 10 | 536,426 | 758,965,359 | 618,290 | 9,762,195 | 0 | 53 | 48 | 111 | 70055 | - |
|  | 15 | 2 | 60 | 76 | 8 | 517,214 | 629,615,563 | 593,155 | 9,284,595 | 0 | 47 | 23 | 100 | 65221 | - |
|  | 15 | 2 | 45 | 61 | 8 | 543,137 | 1,537,128,469 | 606,097 | 11,945,313 | 0 | 55 | 200 | 158 | 48672 | - |
|  | 15 | 2 | 55 | 65 | 5 | 487,200 | 574,426,306 | 545,444 | 8,849,283 | 0 | 39 | 12 | 91 | 49260 | - |
|  | 15 | 2 | 60 | 70 | 5 | 448,983 | 437,705,016 | 512,184 | 7,818,209 | 0 | 28 | -15 | 69 | 52886 | - |
|  | 15 | 2 | 50 | 60 | 5 | 457,516 | 879,019,251 | 511,038 | 9,151,145 | 0 | 30 | 72 | 97 | 41763 | - |
|  | 15 | 2 | 65 | 81 | 8 | 394,528 | 679,425,721 | 470,680 | 7,203,415 | 0* | 12 | 33 | 55 | 60278 | - |
|  | 15 | 2 | 65 | 75 | 5 | 360,621 | 467,362,112 | 426,770 | 6,333,236 | 0* | 3 | -9 | 37 | 51867 | - |
|  | 15 | 2 | 70 | 80 | 5 | 246,441 | 621,060,326 | 311,652 | 4,408,740 | 1* | -30 | 21 | -5 | 45548 | - |
|  | 15 | 3 | 45 | 81 | 12 | 654,996 | 1,668,914,859 | 758,141 | 9,672,427 | 0 | 86 | 226 | 109 | 72921 | - |
|  | 15 | 3 | 50 | 80 | 10 | 622,256 | 1,215,320,565 | 721,865 | 8,633,988 | 0 | 77 | 137 | 86 | 71484 | - |
|  | 15 | 3 | 45 | 75 | 10 | 634,516 | 1,511,280,937 | 728,699 | 9,238,874 | 0 | 81 | 195 | 99 | 67204 | - |
|  | 15 | 3 | 50 | 74 | 8 | 595,642 | 1,048,632,964 | 684,959 | 8,122,393 | 0 | 70 | 105 | 75 | 64642 | - |
|  | 15 | 3 | 50 | 71 | 7 | 569,044 | 970,156,138 | 651,535 | 7,726,530 | 0 | 62 | 89 | 67 | 59587 | - |
|  | 15 | 3 | 45 | 69 | 8 | 581,541 | 1,365,423,262 | 662,702 | 8,491,548 | 0 | 66 | 167 | 83 | 57518 | - |
|  | 15 | 3 | 55 | 79 | 8 | 552,602 | 863,361,916 | 644,772 | 7,287,037 | 0* | 57 | 69 | 57 | 67280 | - |
|  | 15 | 3 | 55 | 76 | 7 | 540,168 | 773,172,576 | 627,024 | 7,025,457 | 0* | 54 | 51 | 52 | 63851 | - |
|  | 15 | 3 | 45 | 66 | 7 | 539,799 | 1,301,237,031 | 613,332 | 7,958,937 | 0 | 54 | 154 | 72 | 51482 | - |
|  | 15 | 3 | 55 | 70 | 5 | 486,313 | 608,400,875 | 559,145 | 6,175,313 | 0* | 38 | 19 | 33 | 53338 | - |
|  | 15 | 3 | 50 | 65 | 5 | 483,671 | 833,700,767 | 550,290 | 6,576,703 | 0* | 38 | 63 | 42 | 46919 | - |
|  | 15 | 3 | 60 | 81 | 7 | 455,233 | 725,558,064 | 542,265 | 5,754,683 | 0* | 30 | 42 | 24 | 62293 | - |
|  | 15 | 3 | 60 | 75 | 5 | 429,959 | 538,913,629 | 506,230 | 5,213,198 | 0* | 22 | 5 | 12 | 55299 | - |
|  | 15 | 3 | 45 | 60 | 5 | 424,329 | 1,165,742,248 | 481,554 | 6,528,285 | 0* | 21 | 128 | 41 | 37810 | - |
|  | 15 | 3 | 60 | 69 | 3 | 360,768 | 372,266,673 | 421,118 | 4,217,763 | 1* | 3 | -27 | -9 | 42697 | - |
|  | 15 | 3 | 50 | 59 | 3 | 349,544 | 687,213,972 | 398,164 | 4,895,446 | 0* | -1 | 34 | 6 | 31661 | - |
|  | 15 | 3 | 65 | 80 | 5 | 330,490 | 611,984,366 | 407,183 | 4,217,313 | 1* | -6 | 20 | -9 | 51943 | - |
|  | 15 | 3 | 65 | 74 | 3 | 294,840 | 410,584,241 | 358,689 | 3,531,481 | 1* | -16 | -20 | -24 | 42955 | - |
|  | 15 | 3 | 45 | 54 | 3 | 277,111 | 958,068,240 | 316,672 | 4,626,028 | 1* | -21 | 87 | 0 | 23437 | - |
|  | 15 | 3 | 70 | 79 | 3 | 205,265 | 553,679,707 | 268,574 | 2,785,162 | 1* | -42 | 8 | -40 | 38809 | - |
|  | 15 | 4 | 45 | 81 | 9 | 565,662 | 1,471,592,383 | 668,389 | 6,610,138 | 0* | 61 | 187 | 43 | 65037 | - |
|  | 15 | 4 | 45 | 77 | 8 | 557,114 | 1,363,847,459 | 653,778 | 6,382,786 | 0* | 59 | 166 | 38 | 61887 | - |
|  | 15 | 4 | 50 | 82 | 8 | 539,523 | 1,148,231,612 | 640,315 | 5,979,136 | 0* | 54 | 124 | 29 | 64797 | - |
|  | 15 | 4 | 50 | 74 | 6 | 514,302 | 930,422,368 | 601,613 | 5,461,626 | 0* | 46 | 82 | 18 | 57237 | - |
|  | 15 | 4 | 45 | 69 | 6 | 500,831 | 1,167,339,795 | 579,731 | 5,697,125 | 0* | 43 | 128 | 23 | 50403 | - |
|  | 15 | 4 | 55 | 79 | 6 | 480,199 | 803,947,912 | 571,221 | 5,018,591 | 0* | 37 | 57 | 8 | 60113 | - |
|  | 15 | 4 | 50 | 70 | 5 | 480,940 | 829,351,044 | 558,268 | 5,064,781 | 0* | 37 | 62 | 9 | 50717 | - |
|  | 15 | 4 | 55 | 75 | 5 | 463,990 | 687,933,364 | 547,314 | 4,716,996 | 0* | 32 | 34 | 2 | 55511 | - |
|  | 15 | 4 | 55 | 71 | 4 | 431,832 | 580,051,619 | 505,190 | 4,300,085 | 1* | 23 | 13 | -7 | 48887 | - |
|  | 15 | 4 | 45 | 65 | 5 | 449,429 | 1,088,037,055 | 517,452 | 5,190,493 | 0* | 28 | 113 | 12 | 42795 | - |
|  | 15 | 4 | 50 | 66 | 4 | 430,390 | 743,983,212 | 496,577 | 4,544,728 | 1* | 23 | 45 | -2 | 42868 | - |
|  | 15 | 4 | 60 | 80 | 5 | 409,263 | 716,240,120 | 495,611 | 4,262,037 | 1* | 16 | 40 | -8 | 57888 | - |
|  | 15 | 4 | 55 | 67 | 3 | 383,196 | 488,753,783 | 445,081 | 3,753,314 | 1* | 9 | -5 | -19 | 40854 | - |
|  | 15 | 4 | 60 | 76 | 4 | 381,041 | 542,421,244 | 457,915 | 3,668,694 | 1* | 8 | 6 | -21 | 50628 | - |
|  | 15 | 4 | 45 | 61 | 4 | 380,945 | 1,009,566,034 | 437,560 | 4,554,086 | 1* | 8 | 97 | -2 | 34476 | - |
|  | 15 | 4 | 60 | 72 | 3 | 350,936 | 427,059,057 | 417,828 | 3,268,939 | 1* | 0 | -17 | -30 | 44028 | - |
|  | 15 | 4 | 50 | 62 | 3 | 361,793 | 665,847,270 | 416,182 | 3,888,870 | 1* | 3 | 30 | -16 | 34122 | - |
|  | 15 | 4 | 65 | 81 | 4 | 290,888 | 623,243,466 | 367,405 | 2,992,424 | 1* | -17 | 22 | -35 | 47367 | - |
|  | 15 | 4 | 65 | 77 | 3 | 279,325 | 493,845,071 | 348,431 | 2,696,038 | 1* | -20 | -4 | -42 | 43293 | - |
|  | 15 | 4 | 45 | 57 | 3 | 300,105 | 914,686,502 | 344,989 | 3,789,925 | 1* | -15 | 79 | -18 | 25950 | - |
|  | 15 | 4 | 70 | 82 | 3 | 188,010 | 629,273,922 | 254,155 | 2,207,244 | 1* | -46 | 23 | -52 | 37695 | - |
|  | 15 | 5 | 45 | 80 | 7 | 500,610 | 1,313,850,021 | 598,861 | 4,935,619 | 0* | 42 | 157 | 6 | 58544 | - |
|  | 15 | 5 | 45 | 75 | 6 | 486,677 | 1,180,942,028 | 576,383 | 4,673,051 | 0* | 39 | 131 | 1 | 54020 | - |
|  | 15 | 5 | 50 | 80 | 6 | 475,679 | 1,015,483,005 | 570,519 | 4,357,665 | 1* | 35 | 98 | -6 | 57598 | - |
|  | 15 | 5 | 50 | 75 | 5 | 461,277 | 878,718,250 | 547,342 | 4,083,026 | 1* | 31 | 72 | -12 | 52912 | - |
|  | 15 | 5 | 45 | 70 | 5 | 452,904 | 1,060,740,744 | 531,053 | 4,315,689 | 1* | 29 | 107 | -7 | 46903 | - |
|  | 15 | 5 | 50 | 70 | 4 | 425,468 | 755,918,185 | 499,406 | 3,709,683 | 1* | 21 | 48 | -20 | 45441 | - |
|  | 15 | 5 | 55 | 80 | 5 | 427,034 | 791,205,827 | 516,150 | 3,678,713 | 1* | 22 | 55 | -21 | 54882 | - |
|  | 15 | 5 | 55 | 75 | 4 | 411,822 | 648,100,598 | 491,754 | 3,390,299 | 1* | 17 | 27 | -27 | 49954 | - |
|  | 15 | 5 | 45 | 65 | 4 | 397,544 | 963,129,558 | 462,149 | 3,850,646 | 1* | 13 | 88 | -17 | 38189 | - |
|  | 15 | 5 | 55 | 70 | 3 | 374,489 | 519,908,398 | 441,748 | 2,996,199 | 1* | 7 | 2 | -35 | 42111 | - |
|  | 15 | 5 | 50 | 65 | 3 | 368,194 | 654,927,801 | 428,023 | 3,220,490 | 1* | 5 | 28 | -31 | 36373 | - |
|  | 15 | 5 | 60 | 80 | 4 | 351,784 | 648,620,717 | 433,826 | 2,968,500 | 1* | 0 | 27 | -36 | 49887 | - |
|  | 15 | 5 | 60 | 75 | 3 | 335,822 | 500,117,407 | 408,271 | 2,661,968 | 1* | -4 | -2 | -43 | 44712 | - |
|  | 15 | 5 | 55 | 65 | 2 | 313,534 | 416,472,620 | 366,077 | 2,467,491 | 1* | -11 | -19 | -47 | 32476 | - |
|  | 15 | 5 | 60 | 70 | 2 | 295,579 | 363,502,321 | 354,389 | 2,231,746 | 1* | -16 | -29 | -52 | 36253 | - |
|  | 15 | 5 | 45 | 60 | 3 | 320,413 | 876,017,339 | 371,037 | 3,261,336 | 1* | -9 | 71 | -30 | 28709 | - |
|  | 15 | 5 | 50 | 60 | 2 | 286,035 | 566,190,578 | 331,010 | 2,592,360 | 1* | -19 | 11 | -44 | 26281 | - |
|  | 15 | 5 | 65 | 80 | 3 | 260,180 | 573,602,400 | 332,360 | 2,262,391 | 1* | -26 | 12 | -51 | 42537 | - |
|  | 15 | 5 | 65 | 75 | 2 | 242,585 | 414,119,308 | 304,378 | 1,928,911 | 1* | -31 | -19 | -58 | 36913 | - |
|  | 15 | 5 | 45 | 55 | 2 | 228,575 | 762,681,838 | 265,001 | 2,533,203 | 1* | -35 | 49 | -45 | 19317 | - |
|  | 15 | 5 | 70 | 80 | 2 | 167,463 | 544,126,708 | 227,634 | 1,604,568 | 1* | -52 | 6 | -65 | 33141 | - |
|  | 20 | 1 | 50 | 80 | 30 | 852,468 | 2,137,772,800 | 936,679 | 31,270,289 | 0 | 143 | 318 | 574 | 90065 | - |
|  | 20 | 1 | 45 | 80 | 35 | 885,735 | 3,034,168,533 | 974,716 | 34,532,939 | 0 | 152 | 493 | 645 | 89853 | - |
|  | 20 | 1 | 50 | 75 | 25 | 831,254 | 1,977,549,582 | 911,352 | 30,529,828 | 0 | 137 | 286 | 558 | 85376 | - |
|  | 20 | 1 | 45 | 75 | 30 | 866,195 | 2,892,364,773 | 951,525 | 33,874,407 | 0 | 147 | 465 | 630 | 85600 | - |
|  | 20 | 1 | 55 | 80 | 25 | 770,114 | 1,463,537,276 | 848,658 | 26,629,041 | 0 | 119 | 186 | 474 | 86886 | - |
|  | 20 | 1 | 50 | 70 | 20 | 783,162 | 1,796,565,424 | 857,576 | 28,791,947 | 0 | 123 | 251 | 521 | 77425 | - |
|  | 20 | 1 | 45 | 70 | 25 | 823,919 | 2,728,765,832 | 904,221 | 32,336,009 | 0 | 135 | 433 | 597 | 78605 | - |
|  | 20 | 1 | 55 | 75 | 20 | 745,519 | 1,285,609,223 | 819,447 | 25,791,141 | 0 | 112 | 151 | 456 | 81503 | - |
|  | 20 | 1 | 55 | 70 | 15 | 690,314 | 1,082,003,696 | 757,769 | 23,771,829 | 0 | 96 | 111 | 413 | 72395 | - |
|  | 20 | 1 | 45 | 65 | 20 | 747,052 | 2,538,769,756 | 820,740 | 29,602,790 | 0 | 113 | 396 | 538 | 68491 | - |
|  | 20 | 1 | 50 | 65 | 15 | 696,137 | 1,586,715,984 | 763,108 | 25,638,880 | 0 | 98 | 210 | 453 | 65967 | - |
|  | 20 | 1 | 60 | 80 | 20 | 628,827 | 1,046,992,635 | 702,983 | 21,013,052 | 0 | 79 | 104 | 353 | 78830 | - |
|  | 20 | 1 | 60 | 75 | 15 | 600,425 | 845,461,430 | 669,287 | 20,034,408 | 0 | 71 | 65 | 332 | 72598 | - |
|  | 20 | 1 | 55 | 65 | 10 | 587,417 | 854,814,872 | 646,616 | 19,986,146 | 0 | 67 | 67 | 331 | 58873 | - |
|  | 20 | 1 | 45 | 60 | 15 | 626,993 | 2,286,176,874 | 692,304 | 25,371,453 | 0 | 78 | 347 | 447 | 55449 | - |
|  | 20 | 1 | 60 | 70 | 10 | 535,732 | 620,132,854 | 597,265 | 17,574,762 | 0 | 52 | 21 | 279 | 61865 | - |
|  | 20 | 1 | 50 | 60 | 10 | 556,406 | 1,310,091,119 | 613,898 | 20,631,714 | 0 | 58 | 156 | 345 | 50837 | - |
|  | 20 | 1 | 65 | 80 | 15 | 459,805 | 824,824,424 | 528,854 | 14,907,893 | 0 | 31 | 61 | 221 | 66734 | - |
|  | 20 | 1 | 65 | 75 | 10 | 425,413 | 598,496,206 | 488,396 | 13,709,428 | 0 | 21 | 17 | 196 | 59277 | - |
|  | 20 | 1 | 45 | 55 | 10 | 463,401 | 1,898,756,805 | 517,279 | 19,430,262 | 0 | 32 | 271 | 319 | 39932 | - |
|  | 20 | 1 | 70 | 80 | 10 | 290,429 | 732,131,472 | 352,853 | 8,897,294 | 0 | -17 | 43 | 92 | 51494 | - |
|  | 20 | 2 | 45 | 81 | 18 | 724,960 | 1,988,589,847 | 831,857 | 14,389,148 | 0 | 106 | 288 | 210 | 79212 | - |
|  | 20 | 2 | 50 | 80 | 15 | 688,245 | 1,421,745,756 | 790,096 | 12,688,793 | 0 | 96 | 178 | 174 | 77462 | - |
|  | 20 | 2 | 45 | 75 | 15 | 701,142 | 1,822,288,770 | 799,504 | 13,880,229 | 0 | 100 | 256 | 199 | 73080 | - |
|  | 20 | 2 | 50 | 76 | 13 | 671,671 | 1,301,914,981 | 767,360 | 12,337,457 | 0 | 91 | 154 | 166 | 73122 | - |
|  | 20 | 2 | 45 | 71 | 13 | 665,798 | 1,714,704,364 | 756,367 | 13,247,347 | 0 | 90 | 235 | 186 | 66590 | - |
|  | 20 | 2 | 55 | 81 | 13 | 614,810 | 1,045,720,282 | 710,900 | 10,765,996 | 0 | 75 | 104 | 132 | 74397 | - |
|  | 20 | 2 | 50 | 70 | 10 | 615,550 | 1,124,458,783 | 698,461 | 11,314,090 | 0 | 75 | 120 | 144 | 62797 | - |
| Initial recommendation (1) | 20 | 2 | 55 | 75 | 10 | 587,514 | 857,478,098 | 673,872 | 10,169,898 | 0 | 67 | 67 | 119 | 67404 | - |
|  | 20 | 2 | 55 | 71 | 8 | 547,167 | 732,966,124 | 624,463 | 9,416,543 | 0 | 56 | 43 | 103 | 59881 | - |
|  | 20 | 2 | 50 | 66 | 8 | 552,972 | 1,016,800,276 | 625,753 | 10,204,053 | 0 | 57 | 99 | 120 | 53825 | - |
|  | 20 | 2 | 45 | 65 | 10 | 577,236 | 1,557,853,051 | 653,501 | 11,711,980 | 0 | 64 | 204 | 153 | 53969 | - |
|  | 20 | 2 | 60 | 80 | 10 | 498,556 | 769,646,702 | 585,738 | 8,330,943 | 0 | 42 | 50 | 80 | 66450 | - |
|  | 20 | 2 | 60 | 76 | 8 | 478,611 | 635,781,678 | 558,864 | 7,909,959 | 0 | 36 | 24 | 71 | 61314 | - |
|  | 20 | 2 | 45 | 61 | 8 | 492,305 | 1,439,698,235 | 557,923 | 10,246,097 | 0 | 40 | 181 | 121 | 44231 | - |
|  | 20 | 2 | 55 | 65 | 5 | 441,059 | 566,241,695 | 501,764 | 7,522,059 | 0 | 26 | 11 | 62 | 44824 | - |
| Age Restriction (2) | 20 | 2 | 60 | 70 | 5 | 409,868 | 444,216,122 | 475,514 | 6,624,968 | 0* | 17 | -13 | 43 | 48722 | - |
|  | 20 | 2 | 50 | 60 | 5 | 409,843 | 837,441,676 | 465,066 | 7,757,511 | 0 | 17 | 64 | 67 | 37442 | - |
|  | 20 | 2 | 65 | 81 | 8 | 366,110 | 691,292,756 | 445,665 | 6,114,863 | 0* | 4 | 35 | 32 | 57065 | - |
|  | 20 | 2 | 65 | 75 | 5 | 331,474 | 474,925,597 | 399,499 | 5,353,641 | 0* | -6 | -7 | 15 | 48311 | - |
|  | 20 | 2 | 45 | 55 | 5 | 331,722 | 1,182,725,297 | 378,856 | 7,378,623 | 0 | -6 | 131 | 59 | 28389 | - |
|  | 20 | 2 | 70 | 80 | 5 | 227,516 | 620,666,855 | 293,741 | 3,680,243 | 1* | -35 | 21 | -21 | 42862 | - |
|  | 20 | 3 | 45 | 81 | 12 | 607,692 | 1,602,741,230 | 715,814 | 8,191,639 | 0 | 73 | 213 | 77 | 69124 | - |
|  | 20 | 3 | 45 | 75 | 10 | 586,140 | 1,435,413,852 | 684,020 | 7,802,609 | 0 | 67 | 180 | 68 | 63012 | - |
|  | 20 | 3 | 50 | 80 | 10 | 573,034 | 1,187,605,873 | 677,014 | 7,245,190 | 0* | 63 | 132 | 56 | 67280 | - |
|  | 20 | 3 | 50 | 74 | 8 | 545,705 | 1,012,911,190 | 638,103 | 6,797,736 | 0* | 55 | 98 | 47 | 60130 | - |
|  | 20 | 3 | 50 | 71 | 7 | 518,994 | 932,863,224 | 603,854 | 6,455,159 | 0* | 48 | 82 | 39 | 54974 | - |
|  | 20 | 3 | 45 | 69 | 8 | 532,373 | 1,284,455,158 | 615,734 | 7,145,554 | 0* | 52 | 151 | 54 | 53061 | - |
|  | 20 | 3 | 55 | 79 | 8 | 507,917 | 860,102,287 | 603,449 | 6,116,034 | 0* | 45 | 68 | 32 | 62996 | - |
|  | 20 | 3 | 55 | 76 | 7 | 495,267 | 765,764,419 | 584,838 | 5,886,280 | 0* | 41 | 50 | 27 | 59427 | - |
|  | 20 | 3 | 45 | 66 | 7 | 491,009 | 1,218,944,942 | 565,913 | 6,679,975 | 0* | 40 | 138 | 44 | 47027 | - |
|  | 20 | 3 | 55 | 70 | 5 | 441,427 | 597,831,530 | 515,566 | 5,149,904 | 0* | 26 | 17 | 11 | 48819 | - |
|  | 20 | 3 | 50 | 65 | 5 | 435,109 | 794,212,550 | 502,525 | 5,467,080 | 0* | 24 | 55 | 18 | 42417 | - |
|  | 20 | 3 | 60 | 81 | 7 | 417,952 | 734,013,070 | 507,384 | 4,801,017 | 0* | 19 | 43 | 4 | 58364 | - |
|  | 20 | 3 | 60 | 75 | 5 | 392,721 | 542,333,555 | 470,338 | 4,332,590 | 1* | 12 | 6 | -7 | 51190 | - |
|  | 20 | 3 | 55 | 64 | 3 | 342,454 | 461,181,246 | 397,826 | 3,965,106 | 1* | -3 | -10 | -14 | 34751 | - |
|  | 20 | 3 | 60 | 69 | 3 | 325,039 | 373,273,737 | 385,321 | 3,483,854 | 1* | -7 | -27 | -25 | 38745 | - |
|  | 20 | 3 | 50 | 59 | 3 | 308,809 | 645,763,766 | 356,699 | 4,037,432 | 1* | -12 | 26 | -13 | 27960 | - |
|  | 20 | 3 | 65 | 80 | 5 | 302,956 | 615,758,970 | 380,449 | 3,476,636 | 1* | -14 | 20 | -25 | 48500 | - |
|  | 20 | 3 | 65 | 74 | 3 | 267,897 | 412,000,819 | 331,476 | 2,894,658 | 1* | -24 | -20 | -38 | 39468 | - |
|  | 20 | 3 | 45 | 54 | 3 | 242,022 | 876,139,858 | 280,040 | 3,813,237 | 1* | -31 | 71 | -18 | 20336 | - |
|  | 20 | 3 | 70 | 79 | 3 | 187,467 | 547,161,952 | 249,992 | 2,295,719 | 1* | -47 | 7 | -50 | 36030 | - |
|  | 20 | 4 | 45 | 81 | 9 | 518,792 | 1,402,355,729 | 623,252 | 5,478,181 | 0* | 48 | 174 | 18 | 60870 | - |
|  | 20 | 4 | 45 | 77 | 8 | 510,013 | 1,290,070,987 | 607,841 | 5,277,742 | 0* | 45 | 152 | 14 | 57568 | - |
|  | 20 | 4 | 50 | 82 | 8 | 492,892 | 1,114,880,456 | 595,250 | 4,951,755 | 0* | 40 | 118 | 7 | 60489 | - |
|  | 20 | 4 | 50 | 74 | 6 | 467,483 | 889,451,220 | 555,250 | 4,500,392 | 1* | 33 | 74 | -3 | 52741 | - |
|  | 20 | 4 | 45 | 69 | 6 | 454,086 | 1,087,994,374 | 532,793 | 4,690,172 | 0* | 29 | 112 | 1 | 45994 | - |
|  | 20 | 4 | 55 | 79 | 6 | 437,789 | 792,559,390 | 529,734 | 4,139,237 | 1* | 25 | 55 | -11 | 55772 | - |
|  | 20 | 4 | 50 | 70 | 5 | 434,544 | 786,324,715 | 511,679 | 4,162,676 | 1* | 24 | 54 | -10 | 46247 | - |
|  | 20 | 4 | 55 | 75 | 5 | 421,753 | 673,983,811 | 505,463 | 3,879,373 | 1* | 20 | 32 | -16 | 51120 | - |
|  | 20 | 4 | 55 | 71 | 4 | 390,248 | 564,980,505 | 463,329 | 3,525,942 | 1* | 11 | 10 | -24 | 44543 | - |
|  | 20 | 4 | 45 | 65 | 5 | 404,356 | 1,006,910,833 | 471,470 | 4,261,725 | 1* | 15 | 97 | -8 | 38577 | - |
|  | 20 | 4 | 50 | 66 | 4 | 385,926 | 700,220,442 | 451,316 | 3,725,365 | 1* | 10 | 37 | -20 | 38629 | - |
|  | 20 | 4 | 60 | 80 | 5 | 374,464 | 717,183,825 | 461,673 | 3,496,816 | 1* | 7 | 40 | -25 | 54025 | - |
|  | 20 | 4 | 55 | 67 | 3 | 343,670 | 473,498,944 | 404,714 | 3,066,955 | 1* | -2 | -8 | -34 | 36785 | - |
|  | 20 | 4 | 60 | 76 | 4 | 346,466 | 537,687,313 | 422,999 | 2,985,970 | 1* | -1 | 5 | -36 | 46645 | - |
|  | 20 | 4 | 60 | 72 | 3 | 317,008 | 422,078,644 | 383,054 | 2,651,791 | 1* | -10 | -18 | -43 | 40116 | - |
|  | 20 | 4 | 45 | 61 | 4 | 339,054 | 929,166,460 | 394,222 | 3,726,084 | 1* | -3 | 81 | -20 | 30612 | - |
|  | 20 | 4 | 50 | 62 | 3 | 321,181 | 622,274,818 | 374,224 | 3,175,748 | 1* | -9 | 22 | -32 | 30312 | - |
|  | 20 | 4 | 65 | 81 | 4 | 265,039 | 620,584,899 | 340,918 | 2,440,677 | 1* | -25 | 21 | -47 | 43923 | - |
|  | 20 | 4 | 65 | 77 | 3 | 253,707 | 490,041,966 | 321,785 | 2,188,537 | 1* | -28 | -4 | -53 | 39852 | - |
|  | 20 | 4 | 45 | 57 | 3 | 264,302 | 834,617,702 | 307,287 | 3,090,030 | 1* | -25 | 63 | -33 | 22711 | - |
|  | 20 | 4 | 70 | 82 | 3 | 170,983 | 617,294,374 | 235,626 | 1,804,350 | 1* | -51 | 21 | -61 | 34896 | - |
|  | 20 | 5 | 45 | 80 | 7 | 455,853 | 1,243,592,264 | 553,786 | 4,020,546 | 1* | 30 | 143 | -13 | 54291 | - |
|  | 20 | 5 | 45 | 75 | 6 | 441,807 | 1,108,012,569 | 530,714 | 3,795,396 | 1* | 26 | 116 | -18 | 49676 | - |
|  | 20 | 5 | 50 | 80 | 6 | 431,501 | 977,626,063 | 525,971 | 3,538,069 | 1* | 23 | 91 | -24 | 53195 | - |
|  | 20 | 5 | 50 | 75 | 5 | 417,301 | 837,930,527 | 502,525 | 3,303,644 | 1* | 19 | 64 | -29 | 48484 | - |
|  | 20 | 5 | 45 | 70 | 5 | 408,630 | 986,434,727 | 485,418 | 3,497,136 | 1* | 16 | 93 | -25 | 42635 | - |
|  | 20 | 5 | 55 | 80 | 5 | 387,642 | 773,856,182 | 476,197 | 2,975,623 | 1* | 10 | 51 | -36 | 50665 | - |
|  | 20 | 5 | 50 | 70 | 4 | 382,544 | 713,081,177 | 455,132 | 2,992,787 | 1* | 9 | 39 | -35 | 41164 | - |
|  | 20 | 5 | 55 | 75 | 4 | 372,872 | 628,937,138 | 451,911 | 2,731,046 | 1* | 6 | 23 | -41 | 45790 | - |
|  | 20 | 5 | 55 | 70 | 3 | 336,584 | 501,385,392 | 402,539 | 2,406,805 | 1* | -4 | -2 | -48 | 38104 | - |
|  | 20 | 5 | 45 | 65 | 4 | 355,563 | 887,926,065 | 418,301 | 3,114,082 | 1* | 1 | 73 | -33 | 34222 | - |
|  | 20 | 5 | 50 | 65 | 3 | 328,183 | 612,000,911 | 386,348 | 2,592,142 | 1* | -7 | 20 | -44 | 32496 | - |
|  | 20 | 5 | 60 | 80 | 4 | 319,227 | 638,731,511 | 400,067 | 2,392,408 | 1* | -9 | 25 | -48 | 46050 | - |
|  | 20 | 5 | 60 | 75 | 3 | 303,575 | 488,947,559 | 374,426 | 2,132,932 | 1* | -14 | -5 | -54 | 40899 | - |
|  | 20 | 5 | 55 | 65 | 2 | 279,206 | 397,977,075 | 330,092 | 1,976,801 | 1* | -21 | -22 | -57 | 28966 | - |
|  | 20 | 5 | 60 | 70 | 2 | 264,818 | 353,748,083 | 321,707 | 1,780,406 | 1* | -25 | -31 | -62 | 32688 | - |
|  | 20 | 5 | 45 | 60 | 3 | 283,754 | 799,465,759 | 332,116 | 2,630,256 | 1* | -19 | 56 | -43 | 25342 | - |
|  | 20 | 5 | 50 | 60 | 2 | 251,933 | 522,159,389 | 294,910 | 2,083,252 | 1* | -28 | 2 | -55 | 23117 | - |
|  | 20 | 5 | 65 | 80 | 3 | 235,818 | 562,593,335 | 306,231 | 1,813,703 | 1* | -33 | 10 | -61 | 39163 | - |
|  | 20 | 5 | 65 | 75 | 2 | 218,843 | 403,992,113 | 278,602 | 1,534,452 | 1* | -38 | -21 | -67 | 33645 | - |
|  | 20 | 5 | 45 | 55 | 2 | 199,724 | 689,374,791 | 233,929 | 2,033,822 | 1* | -43 | 35 | -56 | 16758 | - |
|  | 20 | 5 | 70 | 80 | 2 | 151,559 | 526,025,077 | 209,483 | 1,284,537 | 1* | -57 | 3 | -72 | 30427 | - |
|  | 30 | 1 | 50 | 80 | 30 | 821,429 | 2,156,508,902 | 924,037 | 28,007,745 | 0 | 134 | 321 | 504 | 88939 | - |
|  | 30 | 1 | 45 | 80 | 35 | 861,614 | 3,015,420,600 | 970,052 | 31,244,767 | 0 | 145 | 489 | 574 | 89680 | - |
|  | 30 | 1 | 50 | 75 | 25 | 796,496 | 1,981,667,521 | 893,397 | 27,296,663 | 0 | 127 | 287 | 489 | 83311 | - |
|  | 30 | 1 | 45 | 75 | 30 | 838,437 | 2,855,198,793 | 941,664 | 30,596,676 | 0 | 139 | 458 | 560 | 84505 | - |
|  | 30 | 1 | 55 | 80 | 25 | 737,501 | 1,497,042,428 | 831,821 | 23,638,436 | 0 | 110 | 192 | 410 | 85153 | - |
|  | 30 | 1 | 45 | 70 | 25 | 789,739 | 2,677,339,792 | 885,597 | 29,083,690 | 0 | 125 | 423 | 527 | 76368 | - |
|  | 30 | 1 | 50 | 70 | 20 | 743,143 | 1,789,477,510 | 832,059 | 25,625,686 | 0 | 112 | 249 | 453 | 74360 | - |
|  | 30 | 1 | 55 | 75 | 20 | 709,723 | 1,307,005,465 | 797,834 | 22,852,408 | 0 | 102 | 155 | 393 | 78904 | - |
|  | 30 | 1 | 55 | 70 | 15 | 649,986 | 1,096,698,020 | 729,250 | 20,961,469 | 0 | 85 | 114 | 352 | 68936 | - |
|  | 30 | 1 | 45 | 65 | 20 | 705,865 | 2,475,340,477 | 792,134 | 26,432,829 | 0 | 101 | 383 | 470 | 65249 | - |
|  | 30 | 1 | 50 | 65 | 15 | 650,563 | 1,570,617,520 | 728,818 | 22,649,850 | 0 | 85 | 207 | 388 | 62086 | - |
|  | 30 | 1 | 60 | 80 | 20 | 599,444 | 1,083,990,558 | 686,335 | 18,470,008 | 0 | 71 | 112 | 298 | 76802 | - |
|  | 30 | 1 | 60 | 75 | 15 | 568,154 | 874,202,124 | 648,058 | 17,575,158 | 0 | 62 | 71 | 279 | 69788 | - |
|  | 30 | 1 | 55 | 65 | 10 | 543,570 | 870,248,079 | 611,577 | 17,472,616 | 0 | 55 | 70 | 277 | 54879 | - |
|  | 30 | 1 | 45 | 60 | 15 | 581,588 | 2,211,379,444 | 656,010 | 22,427,299 | 0 | 66 | 332 | 384 | 51667 | - |
|  | 30 | 1 | 60 | 70 | 10 | 499,949 | 649,146,776 | 570,158 | 15,327,192 | 0 | 42 | 27 | 231 | 58353 | - |
|  | 30 | 1 | 50 | 60 | 10 | 510,105 | 1,288,055,672 | 575,166 | 18,033,736 | 0 | 45 | 152 | 289 | 46774 | - |
|  | 30 | 1 | 65 | 80 | 15 | 436,718 | 850,829,582 | 514,628 | 12,959,958 | 0 | 24 | 66 | 179 | 64738 | - |
|  | 30 | 1 | 65 | 75 | 10 | 399,869 | 622,268,209 | 470,060 | 11,886,997 | 0 | 14 | 22 | 156 | 56598 | - |
|  | 30 | 1 | 45 | 55 | 10 | 419,789 | 1,816,904,748 | 478,951 | 16,960,210 | 0 | 19 | 255 | 266 | 36281 | - |
|  | 30 | 1 | 70 | 80 | 10 | 274,335 | 745,348,669 | 342,071 | 7,626,031 | 0 | -22 | 46 | 64 | 49674 | - |
|  | 30 | 2 | 45 | 81 | 18 | 690,163 | 1,962,236,298 | 811,561 | 12,336,037 | 0 | 96 | 283 | 166 | 77354 | - |
|  | 30 | 2 | 50 | 80 | 15 | 650,493 | 1,429,284,981 | 765,424 | 10,781,262 | 0 | 85 | 179 | 132 | 75012 | - |
|  | 30 | 2 | 45 | 75 | 15 | 664,462 | 1,780,535,838 | 775,328 | 11,875,157 | 0 | 89 | 248 | 156 | 70539 | - |
|  | 30 | 2 | 50 | 76 | 13 | 632,610 | 1,301,083,286 | 740,122 | 10,470,691 | 0 | 80 | 154 | 126 | 70243 | - |
|  | 30 | 2 | 45 | 71 | 13 | 627,306 | 1,665,398,540 | 728,625 | 11,305,367 | 0 | 79 | 225 | 144 | 63593 | - |
|  | 30 | 2 | 55 | 81 | 13 | 580,183 | 1,077,242,242 | 688,198 | 9,130,679 | 0 | 65 | 110 | 97 | 71934 | - |
|  | 30 | 2 | 50 | 70 | 10 | 574,408 | 1,115,559,506 | 666,631 | 9,572,835 | 0 | 64 | 118 | 106 | 59310 | - |
|  | 30 | 2 | 55 | 75 | 10 | 551,440 | 879,134,937 | 647,906 | 8,605,265 | 0 | 57 | 72 | 86 | 64381 | - |
|  | 30 | 2 | 55 | 71 | 8 | 509,958 | 750,754,394 | 595,739 | 7,942,711 | 0 | 45 | 47 | 71 | 56544 | - |
|  | 30 | 2 | 45 | 65 | 10 | 536,485 | 1,500,552,285 | 620,361 | 9,942,069 | 0 | 53 | 193 | 114 | 50523 | - |
|  | 30 | 2 | 50 | 66 | 8 | 511,332 | 1,003,830,729 | 591,408 | 8,602,124 | 0 | 46 | 96 | 86 | 50152 | - |
|  | 30 | 2 | 60 | 80 | 10 | 468,365 | 806,004,709 | 564,866 | 6,991,978 | 0* | 33 | 57 | 51 | 63960 | - |
|  | 30 | 2 | 60 | 76 | 8 | 447,648 | 666,088,528 | 535,990 | 6,629,173 | 0* | 27 | 30 | 43 | 58484 | - |
|  | 30 | 2 | 45 | 61 | 8 | 452,061 | 1,376,256,330 | 522,945 | 8,654,539 | 0 | 29 | 169 | 87 | 40752 | - |
|  | 30 | 2 | 55 | 65 | 5 | 404,956 | 578,532,013 | 471,177 | 6,302,288 | 0* | 15 | 13 | 36 | 41462 | - |
|  | 30 | 2 | 50 | 60 | 5 | 372,630 | 816,978,413 | 431,851 | 6,488,748 | 0* | 6 | 60 | 40 | 34105 | - |
|  | 30 | 2 | 65 | 81 | 8 | 342,912 | 723,060,020 | 429,076 | 5,128,835 | 0* | -2 | 41 | 11 | 54791 | - |
|  | 30 | 2 | 65 | 75 | 5 | 307,737 | 501,663,730 | 380,702 | 4,471,702 | 1* | -12 | -2 | -4 | 45682 | - |
|  | 30 | 2 | 45 | 55 | 5 | 298,611 | 1,116,709,025 | 347,950 | 6,172,804 | 0* | -15 | 118 | 33 | 25527 | - |
|  | 30 | 2 | 70 | 80 | 5 | 212,419 | 636,935,832 | 282,176 | 3,042,105 | 1* | -40 | 24 | -34 | 40954 | - |
|  | 30 | 3 | 45 | 81 | 12 | 571,312 | 1,582,020,522 | 690,241 | 6,823,550 | 0* | 63 | 209 | 47 | 66678 | - |
|  | 30 | 3 | 45 | 75 | 10 | 549,025 | 1,403,093,384 | 656,199 | 6,482,618 | 0* | 56 | 174 | 40 | 60176 | - |
|  | 30 | 3 | 50 | 80 | 10 | 536,965 | 1,192,567,897 | 650,613 | 5,978,095 | 0* | 53 | 133 | 29 | 64574 | - |
|  | 30 | 3 | 50 | 74 | 8 | 508,913 | 1,007,716,600 | 609,363 | 5,595,304 | 0* | 45 | 97 | 21 | 57058 | - |
|  | 30 | 3 | 50 | 71 | 7 | 482,100 | 924,248,271 | 573,994 | 5,308,497 | 0* | 37 | 81 | 14 | 51784 | - |
|  | 30 | 3 | 45 | 69 | 8 | 494,506 | 1,242,906,644 | 584,924 | 5,920,596 | 0* | 41 | 143 | 28 | 49881 | - |
|  | 30 | 3 | 55 | 79 | 8 | 474,764 | 884,232,458 | 578,626 | 5,054,431 | 0* | 35 | 73 | 9 | 60163 | - |
|  | 30 | 3 | 55 | 76 | 7 | 461,929 | 785,594,381 | 559,063 | 4,856,016 | 0* | 31 | 53 | 5 | 56423 | - |
|  | 30 | 3 | 45 | 66 | 7 | 453,534 | 1,173,939,824 | 534,339 | 5,525,443 | 0* | 29 | 129 | 19 | 43830 | - |
|  | 30 | 3 | 55 | 70 | 5 | 408,468 | 609,800,187 | 488,270 | 4,231,905 | 1* | 16 | 19 | -9 | 45708 | - |
|  | 30 | 3 | 50 | 65 | 5 | 399,519 | 779,488,505 | 471,727 | 4,481,907 | 1* | 14 | 52 | -3 | 39253 | - |
|  | 30 | 3 | 60 | 81 | 7 | 390,015 | 764,980,767 | 486,404 | 3,949,507 | 1* | 11 | 49 | -15 | 55838 | - |
|  | 30 | 3 | 60 | 75 | 5 | 364,506 | 566,305,643 | 447,694 | 3,550,691 | 1* | 4 | 11 | -23 | 48410 | - |
|  | 30 | 3 | 45 | 60 | 5 | 345,499 | 1,025,467,262 | 405,579 | 4,477,079 | 1* | -2 | 100 | -3 | 30836 | - |
|  | 30 | 3 | 55 | 64 | 3 | 312,866 | 464,666,975 | 371,498 | 3,242,014 | 1* | -11 | -9 | -30 | 31960 | - |
|  | 30 | 3 | 60 | 69 | 3 | 298,580 | 390,857,912 | 362,529 | 2,839,486 | 1* | -15 | -24 | -39 | 36044 | - |
|  | 30 | 3 | 65 | 80 | 5 | 281,256 | 642,805,614 | 363,337 | 2,821,533 | 1* | -20 | 26 | -39 | 46139 | - |
|  | 30 | 3 | 50 | 59 | 3 | 279,287 | 623,678,992 | 329,495 | 3,294,458 | 1* | -21 | 22 | -29 | 25361 | - |
|  | 30 | 3 | 65 | 74 | 3 | 246,693 | 433,903,412 | 313,510 | 2,335,910 | 1* | -30 | -15 | -50 | 37014 | - |
|  | 30 | 3 | 45 | 54 | 3 | 217,246 | 820,101,090 | 256,358 | 3,121,184 | 1* | -38 | 60 | -33 | 18203 | - |
|  | 30 | 3 | 70 | 79 | 3 | 173,815 | 559,350,588 | 238,515 | 1,874,455 | 1* | -51 | 9 | -60 | 34178 | - |
|  | 30 | 4 | 45 | 81 | 9 | 485,172 | 1,381,260,612 | 597,915 | 4,469,030 | 1* | 38 | 170 | -4 | 58339 | - |
|  | 30 | 4 | 45 | 77 | 8 | 476,253 | 1,262,890,869 | 581,654 | 4,296,090 | 1* | 36 | 147 | -7 | 54885 | - |
|  | 30 | 4 | 50 | 82 | 8 | 459,281 | 1,120,375,479 | 569,345 | 4,036,691 | 1* | 31 | 119 | -13 | 57858 | - |
|  | 30 | 4 | 50 | 74 | 6 | 433,635 | 884,373,588 | 527,612 | 3,649,427 | 1* | 23 | 73 | -21 | 49815 | - |
|  | 30 | 4 | 45 | 69 | 6 | 420,441 | 1,049,787,901 | 504,542 | 3,805,979 | 1* | 20 | 105 | -18 | 43107 | - |
|  | 30 | 4 | 55 | 79 | 6 | 407,106 | 812,411,530 | 505,463 | 3,359,131 | 1* | 16 | 59 | -28 | 53008 | - |
|  | 30 | 4 | 50 | 70 | 5 | 401,079 | 776,649,891 | 483,317 | 3,368,980 | 1* | 14 | 52 | -27 | 43271 | - |
|  | 30 | 4 | 55 | 75 | 5 | 391,233 | 689,053,266 | 480,582 | 3,138,590 | 1* | 11 | 35 | -32 | 48273 | - |
|  | 30 | 4 | 55 | 71 | 4 | 360,178 | 576,293,462 | 437,959 | 2,844,056 | 1* | 3 | 13 | -39 | 41676 | - |
|  | 30 | 4 | 45 | 65 | 5 | 372,120 | 964,125,641 | 443,359 | 3,455,207 | 1* | 6 | 88 | -25 | 35783 | - |
|  | 30 | 4 | 50 | 66 | 4 | 354,391 | 685,742,490 | 423,691 | 3,010,991 | 1* | 1 | 34 | -35 | 35802 | - |
|  | 30 | 4 | 60 | 80 | 5 | 348,082 | 742,918,735 | 440,888 | 2,815,030 | 1* | -1 | 45 | -39 | 51481 | - |
|  | 30 | 4 | 60 | 76 | 4 | 320,248 | 558,927,187 | 401,147 | 2,380,326 | 1* | -9 | 9 | -49 | 43954 | - |
|  | 30 | 4 | 55 | 67 | 3 | 315,330 | 480,123,014 | 379,898 | 2,468,629 | 1* | -10 | -6 | -47 | 34088 | - |
|  | 30 | 4 | 60 | 72 | 3 | 291,599 | 439,425,170 | 361,112 | 2,108,221 | 1* | -17 | -14 | -55 | 37473 | - |
|  | 30 | 4 | 45 | 61 | 4 | 309,549 | 881,119,968 | 367,470 | 3,018,571 | 1* | -12 | 72 | -35 | 28060 | - |
|  | 30 | 4 | 50 | 62 | 3 | 292,836 | 602,755,704 | 348,512 | 2,563,145 | 1* | -17 | 18 | -45 | 27796 | - |
|  | 30 | 4 | 65 | 81 | 4 | 245,065 | 644,200,728 | 324,508 | 1,961,845 | 1* | -30 | 26 | -58 | 41672 | - |
|  | 30 | 4 | 65 | 77 | 3 | 234,121 | 510,082,611 | 305,167 | 1,749,797 | 1* | -33 | 0 | -62 | 37576 | - |
|  | 30 | 4 | 45 | 57 | 3 | 239,304 | 782,081,513 | 283,727 | 2,498,539 | 1* | -32 | 53 | -46 | 20563 | - |
|  | 30 | 4 | 70 | 82 | 3 | 158,678 | 627,083,981 | 225,186 | 1,459,539 | 1* | -55 | 22 | -69 | 33200 | - |
|  | 30 | 5 | 45 | 80 | 7 | 423,989 | 1,218,571,847 | 527,834 | 3,216,893 | 1* | 21 | 138 | -31 | 51647 | - |
|  | 30 | 5 | 45 | 75 | 6 | 410,155 | 1,076,287,743 | 504,159 | 3,026,385 | 1* | 17 | 110 | -35 | 46940 | - |
|  | 30 | 5 | 50 | 80 | 6 | 400,903 | 973,934,503 | 500,920 | 2,816,100 | 1* | 14 | 90 | -39 | 50571 | - |
|  | 30 | 5 | 50 | 75 | 5 | 386,707 | 829,028,988 | 476,730 | 2,618,974 | 1* | 10 | 62 | -44 | 45757 | - |
|  | 30 | 5 | 45 | 70 | 5 | 377,314 | 948,600,075 | 458,107 | 2,784,706 | 1* | 7 | 85 | -40 | 39857 | - |
|  | 30 | 5 | 55 | 80 | 5 | 359,767 | 785,705,918 | 453,168 | 2,355,481 | 1* | 2 | 53 | -49 | 48082 | - |
|  | 30 | 5 | 50 | 70 | 4 | 352,598 | 699,827,827 | 428,996 | 2,368,127 | 1* | 0 | 37 | -49 | 38448 | - |
|  | 30 | 5 | 55 | 75 | 4 | 345,140 | 636,414,920 | 428,326 | 2,150,664 | 1* | -2 | 24 | -54 | 43140 | - |
|  | 30 | 5 | 55 | 70 | 3 | 309,785 | 505,081,924 | 378,952 | 1,890,437 | 1* | -12 | -1 | -59 | 35529 | - |
|  | 30 | 5 | 45 | 65 | 4 | 326,293 | 844,769,628 | 391,877 | 2,479,922 | 1* | -7 | 65 | -47 | 31648 | - |
|  | 30 | 5 | 50 | 65 | 3 | 300,208 | 594,348,110 | 361,032 | 2,051,402 | 1* | -15 | 16 | -56 | 29983 | - |
|  | 30 | 5 | 60 | 80 | 4 | 294,955 | 659,360,629 | 379,856 | 1,884,874 | 1* | -16 | 29 | -59 | 43576 | - |
|  | 30 | 5 | 60 | 75 | 3 | 279,756 | 505,329,246 | 353,944 | 1,669,199 | 1* | -20 | -1 | -64 | 38403 | - |
|  | 30 | 5 | 55 | 65 | 2 | 255,084 | 397,186,354 | 308,124 | 1,552,132 | 1* | -27 | -22 | -67 | 26703 | - |
|  | 30 | 5 | 60 | 70 | 2 | 242,720 | 366,222,290 | 302,057 | 1,388,352 | 1* | -31 | -28 | -70 | 30390 | - |
|  | 30 | 5 | 45 | 60 | 3 | 258,297 | 751,697,612 | 308,291 | 2,097,984 | 1* | -26 | 47 | -55 | 23149 | - |
|  | 30 | 5 | 50 | 60 | 2 | 228,359 | 500,061,701 | 272,793 | 1,652,114 | 1* | -35 | -2 | -64 | 21052 | - |
|  | 30 | 5 | 65 | 80 | 3 | 217,507 | 580,727,876 | 290,659 | 1,426,017 | 1* | -38 | 13 | -69 | 37050 | - |
|  | 30 | 5 | 65 | 75 | 2 | 201,048 | 419,752,243 | 262,905 | 1,195,601 | 1* | -43 | -18 | -74 | 31544 | - |
|  | 30 | 5 | 45 | 55 | 2 | 180,286 | 639,903,271 | 215,046 | 1,622,337 | 1* | -49 | 25 | -65 | 15087 | - |
|  | 30 | 5 | 70 | 80 | 2 | 139,987 | 532,381,713 | 199,106 | 1,012,628 | 1* | -60 | 4 | -78 | 28792 | - |
|  | 40 | 1 | 45 | 80 | 35 | 832,619 | 3,052,464,257 | 960,244 | 28,435,120 | 0 | 137 | 496 | 513 | 88954 | - |
|  | 40 | 1 | 50 | 80 | 30 | 789,126 | 2,216,660,058 | 909,514 | 25,324,635 | 0 | 125 | 333 | 446 | 87599 | - |
|  | 40 | 1 | 45 | 75 | 30 | 807,232 | 2,874,636,200 | 928,022 | 27,814,462 | 0 | 130 | 461 | 500 | 83132 | - |
|  | 40 | 1 | 50 | 75 | 25 | 762,211 | 2,025,409,463 | 875,211 | 24,654,523 | 0 | 117 | 296 | 432 | 81370 | - |
|  | 40 | 1 | 55 | 80 | 25 | 705,222 | 1,572,008,525 | 815,670 | 21,201,956 | 0 | 101 | 207 | 357 | 83493 | - |
|  | 40 | 1 | 45 | 70 | 25 | 755,446 | 2,680,188,126 | 866,548 | 26,356,454 | 0 | 115 | 423 | 468 | 74261 | - |
|  | 40 | 1 | 50 | 70 | 20 | 706,080 | 1,818,003,589 | 808,691 | 23,064,263 | 0 | 101 | 255 | 397 | 71760 | - |
|  | 40 | 1 | 55 | 75 | 20 | 675,961 | 1,367,749,639 | 778,528 | 20,470,100 | 0 | 92 | 167 | 341 | 76708 | - |
|  | 40 | 1 | 55 | 70 | 15 | 614,202 | 1,144,177,410 | 705,391 | 18,693,886 | 0 | 75 | 123 | 303 | 66189 | - |
|  | 40 | 1 | 45 | 65 | 20 | 668,489 | 2,461,316,708 | 766,939 | 23,816,360 | 0 | 90 | 381 | 414 | 62553 | - |
|  | 40 | 1 | 50 | 65 | 15 | 612,124 | 1,583,786,993 | 700,862 | 20,254,665 | 0 | 74 | 209 | 337 | 59063 | - |
|  | 40 | 1 | 60 | 80 | 20 | 570,830 | 1,155,392,724 | 670,782 | 16,418,853 | 0 | 62 | 126 | 254 | 75007 | - |
|  | 40 | 1 | 60 | 75 | 15 | 538,114 | 934,065,637 | 629,371 | 15,594,848 | 0 | 53 | 82 | 236 | 67477 | - |
|  | 40 | 1 | 55 | 65 | 10 | 507,610 | 908,056,444 | 584,358 | 15,436,071 | 0 | 44 | 77 | 233 | 51834 | - |
|  | 40 | 1 | 45 | 60 | 15 | 543,137 | 2,181,299,070 | 626,233 | 20,025,718 | 0 | 55 | 326 | 332 | 48674 | - |
|  | 40 | 1 | 60 | 70 | 10 | 468,577 | 700,087,152 | 547,562 | 13,505,046 | 0 | 33 | 37 | 191 | 55595 | - |
|  | 40 | 1 | 50 | 60 | 10 | 472,981 | 1,289,888,754 | 545,047 | 15,949,416 | 0 | 35 | 152 | 244 | 43732 | - |
|  | 40 | 1 | 65 | 80 | 15 | 414,622 | 907,530,530 | 501,976 | 11,392,054 | 0 | 18 | 77 | 146 | 63027 | - |
|  | 40 | 1 | 70 | 80 | 10 | 258,567 | 784,410,431 | 332,341 | 6,591,084 | 0* | -26 | 53 | 42 | 48098 | - |
|  | 40 | 2 | 45 | 81 | 18 | 655,958 | 1,987,993,976 | 791,885 | 10,692,841 | 0 | 87 | 288 | 131 | 75522 | - |
|  | 40 | 2 | 50 | 80 | 15 | 616,114 | 1,476,017,951 | 744,324 | 9,272,862 | 0 | 75 | 188 | 100 | 72889 | - |
|  | 40 | 2 | 45 | 75 | 15 | 629,462 | 1,789,408,620 | 752,968 | 10,275,675 | 0 | 79 | 249 | 122 | 68279 | - |
|  | 40 | 2 | 50 | 76 | 13 | 597,929 | 1,337,974,121 | 717,519 | 8,995,656 | 0 | 70 | 161 | 94 | 67868 | - |
|  | 40 | 2 | 45 | 71 | 13 | 592,013 | 1,664,467,966 | 704,295 | 9,761,274 | 0 | 69 | 225 | 110 | 61069 | - |
|  | 40 | 2 | 55 | 81 | 13 | 546,962 | 1,142,842,270 | 667,229 | 7,823,192 | 0 | 56 | 123 | 69 | 69728 | - |
|  | 40 | 2 | 50 | 70 | 10 | 539,402 | 1,138,526,000 | 641,262 | 8,196,846 | 0 | 54 | 122 | 77 | 56609 | - |
| Planned age expansion (4) | 40 | 2 | 55 | 75 | 10 | 517,847 | 931,519,640 | 624,837 | 7,353,689 | 0 | 47 | 82 | 59 | 61825 | - |
|  | 40 | 2 | 55 | 71 | 8 | 476,397 | 794,499,796 | 571,042 | 6,765,404 | 0* | 36 | 55 | 46 | 53821 | - |
|  | 40 | 2 | 45 | 65 | 10 | 501,981 | 1,482,074,552 | 593,623 | 8,540,432 | 0 | 43 | 189 | 84 | 47830 | - |
|  | 40 | 2 | 50 | 66 | 8 | 477,087 | 1,016,536,462 | 564,701 | 7,336,756 | 0* | 36 | 99 | 58 | 47380 | - |
|  | 40 | 2 | 60 | 80 | 10 | 440,117 | 870,995,011 | 546,537 | 5,921,862 | 0* | 25 | 70 | 28 | 61790 | - |
|  | 40 | 2 | 60 | 76 | 8 | 419,271 | 722,327,245 | 516,347 | 5,603,145 | 0* | 19 | 41 | 21 | 56114 | - |
|  | 40 | 2 | 45 | 61 | 8 | 419,106 | 1,349,638,689 | 495,535 | 7,393,963 | 0 | 19 | 164 | 59 | 38075 | - |
|  | 40 | 2 | 55 | 65 | 5 | 373,877 | 607,865,205 | 445,859 | 5,321,750 | 0* | 6 | 19 | 15 | 38812 | - |
| Current Policy (3) | 40 | 2 | 60 | 70 | 5 | 351,316 | 512,011,709 | 428,784 | 4,637,215 | 0* | 0 | 0 | 0 | 43142 | - |
|  | 40 | 2 | 50 | 60 | 5 | 343,514 | 814,710,211 | 406,885 | 5,486,305 | 0* | -2 | 59 | 18 | 31679 | - |
|  | 40 | 2 | 65 | 81 | 8 | 321,310 | 779,250,054 | 414,845 | 4,319,051 | 1* | -9 | 52 | -7 | 52830 | - |
|  | 40 | 2 | 65 | 75 | 5 | 286,568 | 546,414,128 | 365,124 | 3,740,782 | 1* | -18 | 7 | -19 | 43516 | - |
|  | 40 | 2 | 45 | 55 | 5 | 272,661 | 1,079,412,159 | 324,500 | 5,219,934 | 0* | -22 | 111 | 13 | 23399 | - |
|  | 40 | 2 | 70 | 80 | 5 | 197,579 | 674,327,031 | 271,767 | 2,519,882 | 1* | -44 | 32 | -46 | 39255 | - |
|  | 40 | 3 | 45 | 81 | 12 | 539,089 | 1,602,510,285 | 669,137 | 5,729,585 | 0* | 53 | 213 | 24 | 64622 | - |
|  | 40 | 3 | 45 | 75 | 10 | 516,898 | 1,410,058,606 | 633,712 | 5,429,543 | 0* | 47 | 175 | 17 | 57894 | - |
|  | 40 | 3 | 50 | 80 | 10 | 505,343 | 1,229,592,596 | 628,931 | 4,983,826 | 0* | 44 | 140 | 7 | 62327 | - |
|  | 40 | 3 | 50 | 74 | 8 | 477,375 | 1,032,526,491 | 586,183 | 4,653,247 | 0* | 36 | 102 | 0 | 54582 | - |
|  | 40 | 3 | 50 | 71 | 7 | 451,192 | 941,574,874 | 550,401 | 4,408,724 | 1* | 28 | 84 | -5 | 49282 | - |
|  | 40 | 3 | 45 | 69 | 8 | 463,016 | 1,235,446,436 | 560,816 | 4,945,576 | 0* | 32 | 141 | 7 | 47444 | - |
|  | 40 | 3 | 55 | 79 | 8 | 443,903 | 936,905,948 | 556,889 | 4,198,496 | 1* | 26 | 83 | -9 | 57815 | - |
|  | 40 | 3 | 55 | 76 | 7 | 431,280 | 832,978,007 | 536,853 | 4,025,272 | 1* | 23 | 63 | -13 | 53997 | - |
|  | 40 | 3 | 45 | 66 | 7 | 423,125 | 1,159,224,635 | 510,110 | 4,609,317 | 1* | 20 | 126 | -1 | 41430 | - |
|  | 40 | 3 | 55 | 70 | 5 | 379,329 | 644,186,148 | 465,584 | 3,490,004 | 1* | 8 | 26 | -25 | 43268 | - |
|  | 40 | 3 | 50 | 65 | 5 | 371,057 | 782,842,969 | 448,114 | 3,709,809 | 1* | 6 | 53 | -20 | 36863 | - |
|  | 40 | 3 | 60 | 81 | 7 | 363,893 | 821,934,923 | 467,984 | 3,249,392 | 1* | 4 | 61 | -30 | 53621 | - |
|  | 40 | 3 | 60 | 75 | 5 | 339,074 | 612,208,462 | 428,583 | 2,904,858 | 1* | -3 | 20 | -37 | 46099 | - |
|  | 40 | 3 | 45 | 60 | 5 | 318,675 | 999,157,139 | 382,388 | 3,717,818 | 1* | -9 | 95 | -20 | 28665 | - |
|  | 40 | 3 | 55 | 64 | 3 | 287,464 | 484,951,501 | 350,068 | 2,655,900 | 1* | -18 | -5 | -43 | 29787 | - |
|  | 40 | 3 | 60 | 69 | 3 | 275,394 | 423,943,758 | 343,569 | 2,305,419 | 1* | -22 | -17 | -50 | 33857 | - |
|  | 40 | 3 | 65 | 80 | 5 | 261,622 | 688,879,418 | 349,142 | 2,294,817 | 1* | -26 | 35 | -51 | 44194 | - |
|  | 40 | 3 | 50 | 59 | 3 | 256,930 | 615,085,390 | 309,518 | 2,712,412 | 1* | -27 | 20 | -42 | 23456 | - |
|  | 40 | 3 | 65 | 74 | 3 | 228,254 | 468,104,170 | 299,000 | 1,882,127 | 1* | -35 | -9 | -59 | 35081 | - |
|  | 40 | 3 | 45 | 54 | 3 | 197,941 | 787,461,947 | 238,524 | 2,578,134 | 1* | -44 | 54 | -44 | 16635 | - |
|  | 40 | 3 | 70 | 79 | 3 | 160,501 | 590,579,766 | 228,384 | 1,517,950 | 1* | -54 | 15 | -67 | 32544 | - |
|  | 40 | 4 | 45 | 81 | 9 | 454,871 | 1,399,822,326 | 576,431 | 3,666,678 | 1* | 29 | 173 | -21 | 56230 | - |
|  | 40 | 4 | 45 | 77 | 8 | 446,142 | 1,274,045,233 | 559,629 | 3,515,991 | 1* | 27 | 149 | -24 | 52677 | - |
|  | 40 | 4 | 50 | 82 | 8 | 429,797 | 1,156,866,635 | 548,134 | 3,302,982 | 1* | 22 | 126 | -29 | 55628 | - |
|  | 40 | 4 | 50 | 74 | 6 | 404,790 | 906,502,122 | 505,491 | 2,968,012 | 1* | 15 | 77 | -36 | 47454 | - |
|  | 40 | 4 | 45 | 69 | 6 | 391,619 | 1,044,827,528 | 481,638 | 3,106,093 | 1* | 11 | 104 | -33 | 40834 | - |
|  | 40 | 4 | 55 | 79 | 6 | 378,738 | 857,241,491 | 484,301 | 2,724,363 | 1* | 8 | 67 | -41 | 50658 | - |
|  | 40 | 4 | 50 | 70 | 5 | 373,297 | 790,728,136 | 461,122 | 2,735,129 | 1* | 6 | 54 | -41 | 40946 | - |
|  | 40 | 4 | 55 | 75 | 5 | 363,497 | 726,111,151 | 459,209 | 2,536,331 | 1* | 3 | 42 | -45 | 45918 | - |
|  | 40 | 4 | 55 | 71 | 4 | 333,297 | 606,427,033 | 416,418 | 2,290,112 | 1* | -5 | 18 | -51 | 39331 | - |
|  | 40 | 4 | 45 | 65 | 5 | 345,315 | 950,079,607 | 421,098 | 2,817,972 | 1* | -2 | 86 | -39 | 33642 | - |
|  | 40 | 4 | 50 | 66 | 4 | 328,628 | 691,451,211 | 402,261 | 2,442,137 | 1* | -6 | 35 | -47 | 33626 | - |
|  | 40 | 4 | 60 | 80 | 5 | 324,078 | 792,361,179 | 423,436 | 2,271,907 | 1* | -8 | 55 | -51 | 49378 | - |
|  | 40 | 4 | 60 | 76 | 4 | 296,952 | 598,975,325 | 383,099 | 1,896,691 | 1* | -15 | 17 | -59 | 41814 | - |
|  | 40 | 4 | 55 | 67 | 3 | 290,608 | 502,059,880 | 359,287 | 1,981,724 | 1* | -17 | -2 | -57 | 31916 | - |
|  | 40 | 4 | 60 | 72 | 3 | 269,132 | 473,091,308 | 342,921 | 1,672,010 | 1* | -23 | -8 | -64 | 35353 | - |
|  | 40 | 4 | 45 | 61 | 4 | 285,804 | 858,098,279 | 346,857 | 2,461,120 | 1* | -19 | 68 | -47 | 26182 | - |
|  | 40 | 4 | 50 | 62 | 3 | 269,963 | 600,842,580 | 328,659 | 2,076,177 | 1* | -23 | 17 | -55 | 25873 | - |
|  | 40 | 4 | 65 | 81 | 4 | 227,009 | 684,666,986 | 310,930 | 1,566,126 | 1* | -35 | 34 | -66 | 39810 | - |
|  | 40 | 4 | 65 | 77 | 3 | 216,524 | 545,092,940 | 291,432 | 1,384,721 | 1* | -38 | 6 | -70 | 35709 | - |
|  | 40 | 4 | 45 | 57 | 3 | 219,256 | 753,879,981 | 265,576 | 2,035,451 | 1* | -38 | 47 | -56 | 18961 | - |
|  | 40 | 4 | 70 | 82 | 3 | 146,274 | 656,767,988 | 215,686 | 1,167,618 | 1* | -58 | 28 | -75 | 31655 | - |
|  | 40 | 5 | 45 | 80 | 7 | 395,518 | 1,230,204,791 | 506,268 | 2,585,504 | 1* | 13 | 140 | -44 | 49495 | - |
|  | 40 | 5 | 45 | 75 | 6 | 382,070 | 1,080,653,063 | 482,210 | 2,423,387 | 1* | 9 | 111 | -48 | 44749 | - |
|  | 40 | 5 | 50 | 80 | 6 | 373,785 | 999,592,131 | 480,185 | 2,246,826 | 1* | 6 | 95 | -52 | 48377 | - |
|  | 40 | 5 | 50 | 75 | 5 | 360,307 | 847,525,345 | 455,966 | 2,080,270 | 1* | 3 | 66 | -55 | 43579 | - |
|  | 40 | 5 | 45 | 70 | 5 | 350,153 | 944,507,885 | 435,932 | 2,226,717 | 1* | 0 | 84 | -52 | 37694 | - |
|  | 40 | 5 | 55 | 80 | 5 | 333,703 | 824,410,604 | 433,204 | 1,864,114 | 1* | -5 | 61 | -60 | 45884 | - |
|  | 40 | 5 | 50 | 70 | 4 | 326,964 | 710,812,937 | 407,923 | 1,876,218 | 1* | -7 | 39 | -60 | 36286 | - |
|  | 40 | 5 | 55 | 75 | 4 | 319,785 | 668,397,221 | 408,296 | 1,691,016 | 1* | -9 | 31 | -64 | 40948 | - |
|  | 40 | 5 | 45 | 65 | 4 | 301,780 | 830,958,436 | 371,026 | 1,986,417 | 1* | -14 | 62 | -57 | 29684 | - |
|  | 40 | 5 | 55 | 70 | 3 | 285,855 | 528,857,979 | 359,263 | 1,481,388 | 1* | -19 | 3 | -68 | 33443 | - |
|  | 40 | 5 | 50 | 65 | 3 | 277,105 | 596,751,993 | 341,253 | 1,625,619 | 1* | -21 | 17 | -65 | 28041 | - |
|  | 40 | 5 | 60 | 80 | 4 | 273,236 | 698,814,185 | 362,956 | 1,480,334 | 1* | -22 | 36 | -68 | 41518 | - |
|  | 40 | 5 | 60 | 75 | 3 | 258,702 | 539,048,464 | 337,011 | 1,298,675 | 1* | -26 | 5 | -72 | 36378 | - |
|  | 40 | 5 | 55 | 65 | 2 | 234,236 | 410,994,541 | 290,203 | 1,214,760 | 1* | -33 | -20 | -74 | 24897 | - |
|  | 40 | 5 | 60 | 70 | 2 | 223,496 | 392,251,527 | 285,946 | 1,073,511 | 1* | -36 | -23 | -77 | 28537 | - |
|  | 40 | 5 | 45 | 60 | 3 | 237,425 | 729,472,599 | 289,707 | 1,683,848 | 1* | -32 | 42 | -64 | 21477 | - |
|  | 40 | 5 | 50 | 60 | 2 | 209,762 | 493,166,843 | 256,132 | 1,312,452 | 1* | -40 | -4 | -72 | 19507 | - |
|  | 40 | 5 | 65 | 80 | 3 | 200,769 | 613,901,647 | 277,464 | 1,112,573 | 1* | -43 | 20 | -76 | 35246 | - |
|  | 40 | 5 | 65 | 75 | 2 | 185,289 | 447,436,913 | 250,036 | 920,320 | 1* | -47 | -13 | -80 | 29815 | - |
|  | 40 | 5 | 45 | 55 | 2 | 164,407 | 611,938,881 | 200,307 | 1,304,021 | 1* | -53 | 20 | -72 | 13838 | - |
|  | 40 | 5 | 70 | 80 | 2 | 128,496 | 556,541,437 | 189,832 | 789,794 | 1* | -63 | 9 | -83 | 27297 | - |

*= within the future extended capacity
